# Optimization and Metabolomic Profiling of *Wolffia globosa* Extract Reveal Multitarget Neuroprotective Activity Against Alzheimer’s Disease with In Vitro, In Silico, and In Vivo Validation

**DOI:** 10.3390/ijms27188266

**Published:** 2026-09-17

**Authors:** Piya Temviriyanukul, Woorawee Inthachat, Tanongsak Laowanitwattana, Pensiri Buacheen, Uthaiwan Suttisansanee, Pornsiri Pitchakarn

**Affiliations:** 1 Food and Nutrition Academic and Research Cluster, Institute of Nutrition, Mahidol University, Nakhon Pathom 73170, Thailand; piya.tem@mahidol.ac.th (P.T.); woorawee.int@mahidol.ac.th (W.I.); uthaiwan.sut@mahidol.ac.th (U.S.); 2 Department of Biochemistry, Faculty of Medicine, Chiang Mai University, Chiang Mai 50200, Thailand; tanongsak.l@cmu.ac.th (T.L.); pensiri.bua@cmu.ac.th (P.B.)

**Keywords:** antioxidants, donepezil, enzyme inhibition, extraction optimization, metabolomics, molecular docking, polyphenols, synergism, *Wolffia globosa*

## Abstract

*Wolffia globosa*, the world’s smallest flowering plant, is a sustainable protein- and phytochemical-rich food source with emerging neuroprotective potential. This study presents the first comprehensive characterization that integrates extraction optimization, multitarget bioactivity profiling, drug synergy assessment, high-resolution metabolomics, molecular docking, and in vivo validation in *Drosophila melanogaster*. Ethanol-based extraction was optimized using a Box–Behnken design and response surface methodology (RSM), yielding optimal conditions of 32% ethanol, 1:40 (g/mL) solid-to-solvent ratio, 60 °C, and 28 min. The optimized extract exhibited a total phenolic content (TPC) of 15.17 ± 0.27 mg GAE/g DW, a total flavonoid content (TFC) of 22.34 ± 1.71 mg QE/g DW, and an exceptional ORAC antioxidant activity of 4374.33 ± 395.65 µmol TE/g DW. Potent and selective BACE-1 inhibition was observed (IC_50_: 1.59 ± 0.11 mg/mL), alongside moderate AChE (IC_50_: 7.42 mg/mL) and BChE (IC_50_: 6.55 mg/mL) inhibition. Synergistic interactions with donepezil were confirmed by the Chou–Talalay combination index method, with combined AChE, BChE, and BACE-1 inhibitions reaching 60.3%, 77.0%, and 82.2%, respectively—substantially exceeding theoretical additive values. Multi-platform metabolite profiling by HPLC-QTOF-MS/MS and LC-ESI-MS/MS identified luteolin, apigenin, caffeic acid, isovitexin, schaftoside, and pinolenic acid as the principal bioactives. Molecular docking predicted favorable binding of luteolin (delta-G: −10.24 kcal/mol, AChE) and naringenin (delta-G: −8.76 kcal/mol, BACE-1) against crystal structures of human AChE (PDB: 7E3H), BChE (PDB: 4TPK), and BACE-1 (PDB: 6EQM). In vivo, the *W. globosa* extract significantly improved locomotor performance and suppressed brain BACE-1 activity in an *Drosophila* AD model over 28 days. These findings establish *W. globosa* as a multifunctional nutraceutical candidate for neurodegenerative disease prevention.

## 1. Introduction

The demands of a growing global population and accelerating climate change underscore the urgent need for novel, sustainable, and nutritionally dense food sources [1,2]. Conventional protein crops such as soy occupy vast arable land and carry associated allergenicity concerns, driving intensified interest in alternative plant-based ingredients [3]. Aquatic weeds belonging to the family Lemnaceae, commonly referred to as duckweeds, are attracting significant scientific attention as a feasible biomass resource owing to their exceptionally swift growth rates, elevated protein content, and adaptation to resource-efficient, low-footprint cultivation systems [1,4,5,6]. Among the five recognized genera, *Spirodela*, *Landoltia*, *Lemna*, *Wolffiella*, and *Wolffia*, species of the genus *Wolffia* are uniquely edible and have long been consumed as a traditional food in Southeast Asia, particularly Thailand [5,6,7,8].

*Wolffia globosa* (Roxb.) Hartog & Plas, designated as the smallest known flowering plant on Earth, exemplifies the remarkable nutritional potential of the Lemnaceae family. Biochemical analyses across multiple cultivation systems have documented a protein content ranging from 29.6 to 48.2% of dry weight [7,8,9,10]. Beyond macronutrient density, *W. globosa* harbors an abundant repertoire of secondary metabolites, including phenolic acids, flavonoids, anthocyanins, and carotenoids [7,9,10,11]. Key bioactive molecules identified in ethanolic extracts include beta-carotene, ferulic acid, luteolin-7-*O*-β-d-glucoside, and kaempferol, which collectively confer significant free radical scavenging capacity, as evidenced by DPPH inhibition reaching 75.77 ± 0.93% at 1000 µg/mL in freshly prepared extracts [12]. The phenolic and flavonoid profiles of *W. globosa* are influenced by cultivation conditions and environmental factors, with farmed specimens consistently exhibiting higher total phenolic content (TPC) and total flavonoid content (TFC) relative to wild-harvested counterparts, underscoring the critical role of optimized production systems in maximizing bioactive yields [7,10].

These characteristics collectively establish *W. globosa* as a compelling sustainable food source that simultaneously offers high nutritional value and diverse bioactive properties. Its rapid biomass doubling time, reported at 32–109 h under favorable conditions, combined with low land and water requirements, positions it as a viable ingredient for functional food formulation with both nutritional and health-promoting dimensions. Recent research has further underscored the medicinal potential of *W. globosa*, with extracts demonstrating antioxidant, anti-inflammatory, anti-diabetic, and lipid-lowering activities [7,9,10,11]. These properties correspond with the escalating global demand for functional foods and nutraceuticals capable of mitigating the burden of chronic diseases, including neurological disorders [3].

The global incidence of Alzheimer’s disease (AD) is escalating in direct correlation with aging populations worldwide. According to international epidemiological estimates, tens of millions of people are currently living with dementia, with Alzheimer’s disease accounting for 60–70% of cases and generating approximately 10 million new cases annually. Projections indicate that these figures will rise dramatically in the coming decades, with the number of people living with dementia expected to reach around 78 million by 2030 and 139 million by 2050, with the most rapid growth anticipated in low- and middle-income countries across Asia, South Asia, and the Western Pacific region [13,14,15]. The global economic burden of dementia already exceeds trillions of US dollars annually, with costs projected to increase substantially, highlighting the profound socioeconomic urgency of developing effective prevention and treatment strategies [16]. The pathophysiology of AD is multifactorial and complex, involving intersecting mechanisms including cholinergic impairment, accumulation of amyloid-β (Aβ) plaques, neurofibrillary tangles, oxidative stress, and neuroinflammation [17,18]. Current pharmacological approaches, including acetylcholinesterase (AChE) inhibitors, donepezil, rivastigmine, and galantamine, as well as the NMDA receptor antagonist memantine, provide only symptomatic relief without modifying the underlying disease course [19,20].

In the past decade, dietary polyphenols and flavonoids have emerged as a prominent area of investigation in food chemistry and neuropharmacology, attracting considerable interest due to their capacity to engage with multiple molecular targets implicated in AD pathology [21,22,23,24,25]. Unlike conventional single-target pharmacological agents, plant-derived polyphenols frequently exert their neuroprotective effects through multitarget mechanisms, including activation of the Nrf2/ARE pathway to counter oxidative stress, inhibition of NF-κB-mediated neuroinflammation, suppression of Aβ aggregation, and inhibition of key enzymatic pathways. Specific bioactive compounds found in *W. globosa* include luteolin, apigenin, caffeic acid, and quercetin [26]. These phytochemicals have been individually demonstrated to inhibit neurotoxic enzymes, including AChE, BChE, and beta-secretase 1 (BACE-1), the rate-limiting enzyme responsible for cleaving amyloid precursor protein (APP) to generate Aβ peptides, a pathological hallmark of AD. Consequently, these compounds may target both symptomatic manifestations and upstream pathogenic mechanisms of AD [21,27,28,29]. Apigenin and luteolin, in particular, modulate microglial activation via the suppression of CD40 expression and STAT1 phosphorylation, reducing the release of pro-inflammatory cytokines including TNF-α and IL-6 in neuronal environments [28,29,30]. Caffeic acid has been identified as among the most potent naturally occurring neuroprotective phenolic acids, demonstrating substantial protection against neurotoxin-induced injury in neuronal cell models [29,31]. The presence of these compounds in *W. globosa* thus provides a mechanistic basis for evaluating its extract as a multitarget neuroprotective candidate. In addition to AD-specific targets, there is growing evidence that metabolic dysfunction (type 2 diabetes, insulin resistance, dyslipidemia) is a potentially modifiable risk factor for AD, leading to the proposed concept of AD as “type 3 diabetes” [32]. As α-amylase and α-glucosidase are key enzymes for carbohydrate digestion and postprandial glycemic regulation and pancreatic lipase is a key enzyme for dietary fat digestion and absorption, the W. globosa extract was also screened against these enzymes to investigate whether it had any ancillary metabolic activities that could complement its AD-directed effects [33].

A particularly novel and promising direction in nutraceutical–pharmacological integration involves the assessment of synergistic interactions between plant extract bioactives and established synthetic drugs [34]. Synergistic combinations allow for reductions in the effective therapeutic dose of pharmacological agents, thereby minimizing adverse effects while preserving or enhancing efficacy [35]. Precedent for this strategy is established by studies demonstrating synergistic or additive AChE, BChE, and BACE-1 inhibition when plant-derived compounds such as quercetin and kaempferol are combined with donepezil, as reported in investigations of *Diplazium esculentum* [36]. Similarly, *Ginkgo biloba* combined with donepezil has demonstrated superior anti-amnestic and procholinergic effects compared with either agent alone [37]. These findings indicate that the combination of *W. globosa* extracts with donepezil represents a scientifically grounded and translationally relevant strategy for the evidence-based integration of dietary and pharmacological approaches to AD therapy [34,35,36,37].

Despite the broad nutritional and bioactive attributes of *W. globosa*, systematic characterization of its extract under optimized conditions and comprehensive evaluation of its multitarget biological activities, remains limited. Extraction solvent polarity, temperature, time, and solid-to-liquid ratio profoundly influence the yield and composition of recovered bioactive metabolites, necessitating a rigorous optimization framework [38,39,40]. Response surface methodology (RSM) employing Box–Behnken design (BBD) has been validated as a robust and efficient statistical approach for the simultaneous optimization of multiple extraction parameters, minimizing experimental runs while generating predictive models of phenolic and flavonoid recovery [38,39,40,41]. Comprehensive metabolite profiling using combined HPLC-qTOF-MS and LC-ESI-MS/MS enables detailed identification and semi-quantitative characterization of both polar and non-polar phytochemical fractions, facilitating linkage between chemical composition and observed biological activities [42]. Molecular docking studies further provide mechanistic insights into the specific binding interactions between identified bioactive molecules and target enzyme active sites, supporting computational validation of in vitro observations [43].

## 2. Results

### 2.1. Effect of Extraction Solvent on TPCs and TFCs

Solvent polarity exerted a decisive effect on bioactive recovery (Table 1). In direct filtrate preparations, 70% ethanol (TPCs: 15.68 ± 0.08 mg GAE/g DW; TFCs: 25.03 ± 0.84 mg QE/g DW) and distilled water (TPCs: 14.73 ± 0.23 mg GAE/g DW; TFCs: 21.34 ± 0.74 mg QE/g DW) both yielded detectable phenolic and flavonoid contents, while hexane and dichloromethane extracts produced precipitation artifacts and were classified as not detected (ND). Following rotary evaporation and re-dissolution in DMSO, 70% ethanol yielded the highest TPCs (16.38 ± 0.43 mg GAE/g DW) and TFCs (27.14 ± 0.58 mg QE/g DW), significantly exceeding distilled water (TPCs: 6.97 ± 0.92 mg GAE/g DW; TFCs: 20.12 ± 0.84 mg QE/g DW), while hexane (TPCs: 0.98 ± 0.09 mg GAE/g DW) and dichloromethane (TPCs: 0.68 ± 0.06 mg GAE/g DW) provided only trace phenolics and no detectable flavonoids. Accordingly, 70% ethanol was selected as the extraction solvent for all subsequent optimization experiments.

### 2.2. Effect of Extraction Ratio, Temperature and Extraction Time on TPCs and TFCs 

Single-factor experiments were performed to define appropriate ranges for multivariate optimization for BBD and RSM.

Ethanol concentration: TPCs peaked at 60% ethanol (12.72 ± 0.32 mg GAE/g DW), whereas TFCs increased progressively to its highest value at 80% ethanol (13.59 ± 3.00 mg QE/g DW), with 60% and 40% ethanol also yielding significantly higher TFCs than pure water (9.45 ± 0.69 mg QE/g DW) (Table 1). These findings indicate that intermediate-to-high ethanol strengths favor flavonoid recovery, while maximal phenolic extraction occurs at 60% ethanol under the tested conditions.

Solid-to-solvent ratio: TPCs were highest at a ratio of 1:50 (13.04 ± 0.11 mg GAE/g DW), whereas TFCs did not differ significantly between 1:30 (10.40 ± 1.04 mg QE/g DW) and 1:40 (10.55 ± 0.80 mg QE/g DW), but declined at 1:50 (8.23 ± 0.50 mg QE/g DW) (Table 2). This suggests a trade-off at high solvent volumes, where increased dilution enhances phenolic extraction but may reduce flavonoid concentration in the extract.

Temperature: TFCs increased markedly from 14.59 ± 0.45 mg QE/g DW at 30 °C to 19.75 ± 0.77 mg QE/g DW at 70 °C, whereas TPCs values were comparatively less temperature-sensitive, remaining within 12.13–12.51 mg GAE/g DW across the examined range (Table 3). Elevated temperatures thus preferentially enhanced flavonoid extraction without substantially affecting the total phenolic content.

Extraction time: TPCs were highest at 45 min (12.27 ± 0.06 mg GAE/g DW), while TFCs peaked at 60 min (14.08 ± 0.40 mg QE/g DW) (Table 4). Shorter extraction times (15–30 min) yielded lower TPCs and TFCs, indicating that extended extraction up to 45–60 min is required to approach the maximal recovery of phenolics and flavonoids under these conditions. Accordingly, 70% ethanol was selected for all subsequent optimization experiments.

### 2.3. RSM Optimization Using Box–Behnken Design

#### 2.3.1. BBD Matrix and Regression Models

The ranges of extraction factors obtained from the above results were subjected for BBD and resulted in 27 runs, as shown in Table 5. After the completion of all runs, TPC values ranged from 7.75 ± 0.27 mg GAE/g DW (Run 17: 70% ethanol, 1:30 g/mL, 45 °C, 20 min) to 14.30 ± 0.19 mg GAE/g DW (Run 16: 30% ethanol, 1:30 g/mL, 60 °C, 40 min). TFC values ranged from 9.32 ± 0.67 mg QE/g DW (Run 17) to 23.55 ± 0.70 mg QE/g DW (Run 16). These results indicate that relatively low ethanol concentration (30%) combined with moderate solid-to-solvent ratio (1:30 g/mL) and elevated temperature (60 °C) promotes the simultaneous enhancement of phenolic and flavonoid extraction within the tested ranges.

The experimental TPC and TFC data were fitted to second-order polynomial models of the form:

$$\mathrm{TPCs}\;\mathrm{or}\;\mathrm{TFCs}=\beta_{0}+\sum \beta_{i}X_{i}+\sum \beta_{ij}X_{i}X_{j}+\sum \beta_{ii}X_{i}^{2}$$

where $X_{1}$, $X_{2}$, $X_{3}$, and $X_{4}$ represent ethanol concentration (% *v*/*v*), solid-to-solvent ratio (g/mL), extraction temperature (°C), and extraction time (min), respectively.

The fitted equations in uncoded form were:

$$\begin{array}{cc} TPCs & =38.4549-0.4241X_{1}-0.3868X_{2}-0.4434X_{3}-0.1451X_{4}\\ & +0.0026X_{1}X_{2}+0.0007X_{1}X_{3}+0.0013X_{1}X_{4}+0.0045X_{2}X_{3}\\ & -0.0002X_{2}X_{4}+0.0017X_{3}X_{4}+0.0017X_{1}^{2}+0.0028X_{2}^{2}+0.0027X_{3}^{2}+0.0003X_{4}^{2} \end{array}$$

$$\begin{array}{cc} \mathrm{TFCs} & =-11.1801+0.7795X_{1}+0.8549X_{2}-0.1757X_{3}+0.2867X_{4}\\ & -0.0002X_{1}X_{2}-0.0018X_{1}X_{3}+0.0003X_{1}X_{4}+0.0069X_{2}X_{3}\\ & -0.0035X_{2}X_{4}-0.0011X_{3}X_{4}-0.0092X_{1}^{2}-0.0157X_{2}^{2}+0.0009X_{3}^{2}-0.0017X_{4}^{2} \end{array}$$

**Figure 1:**
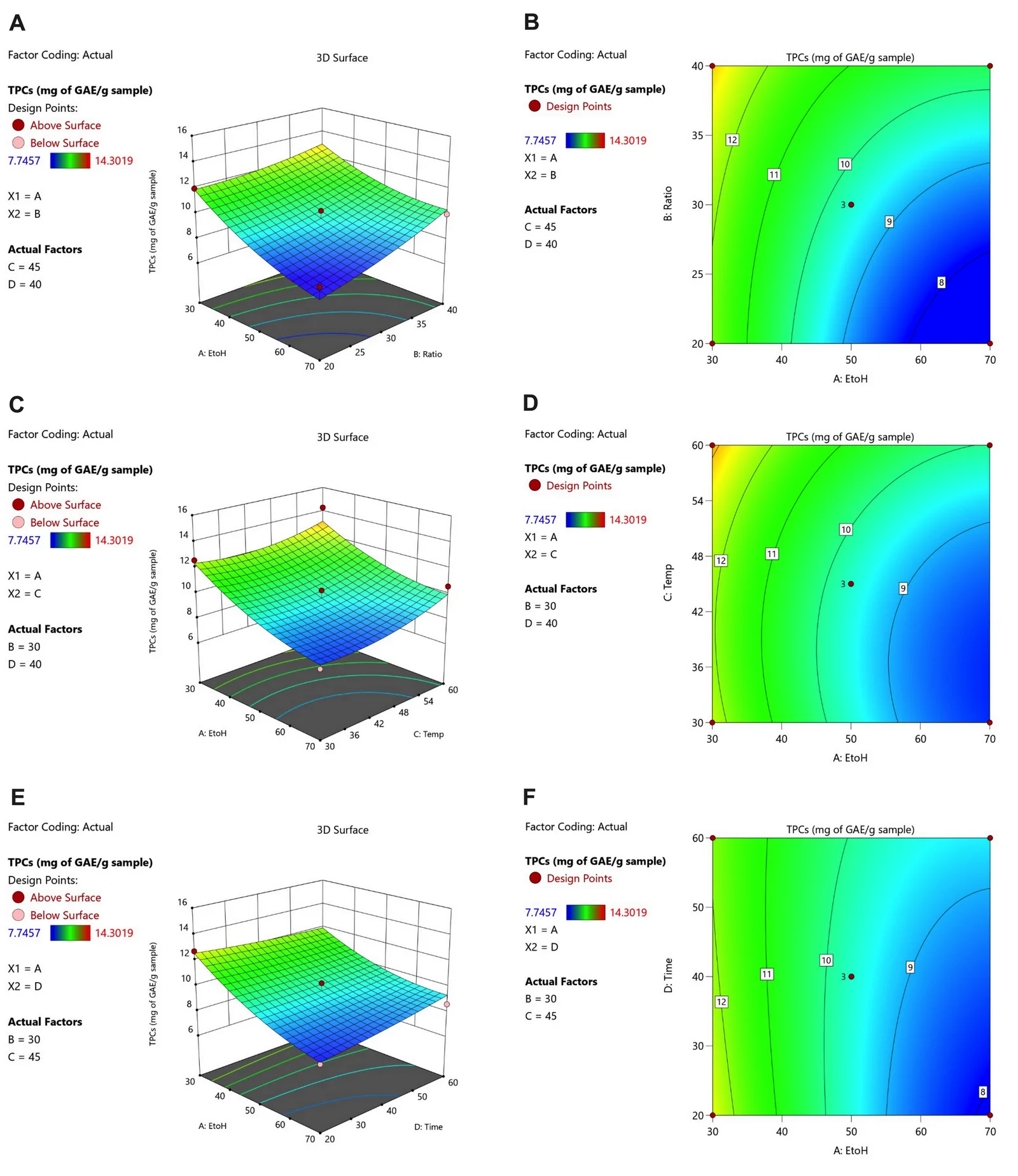

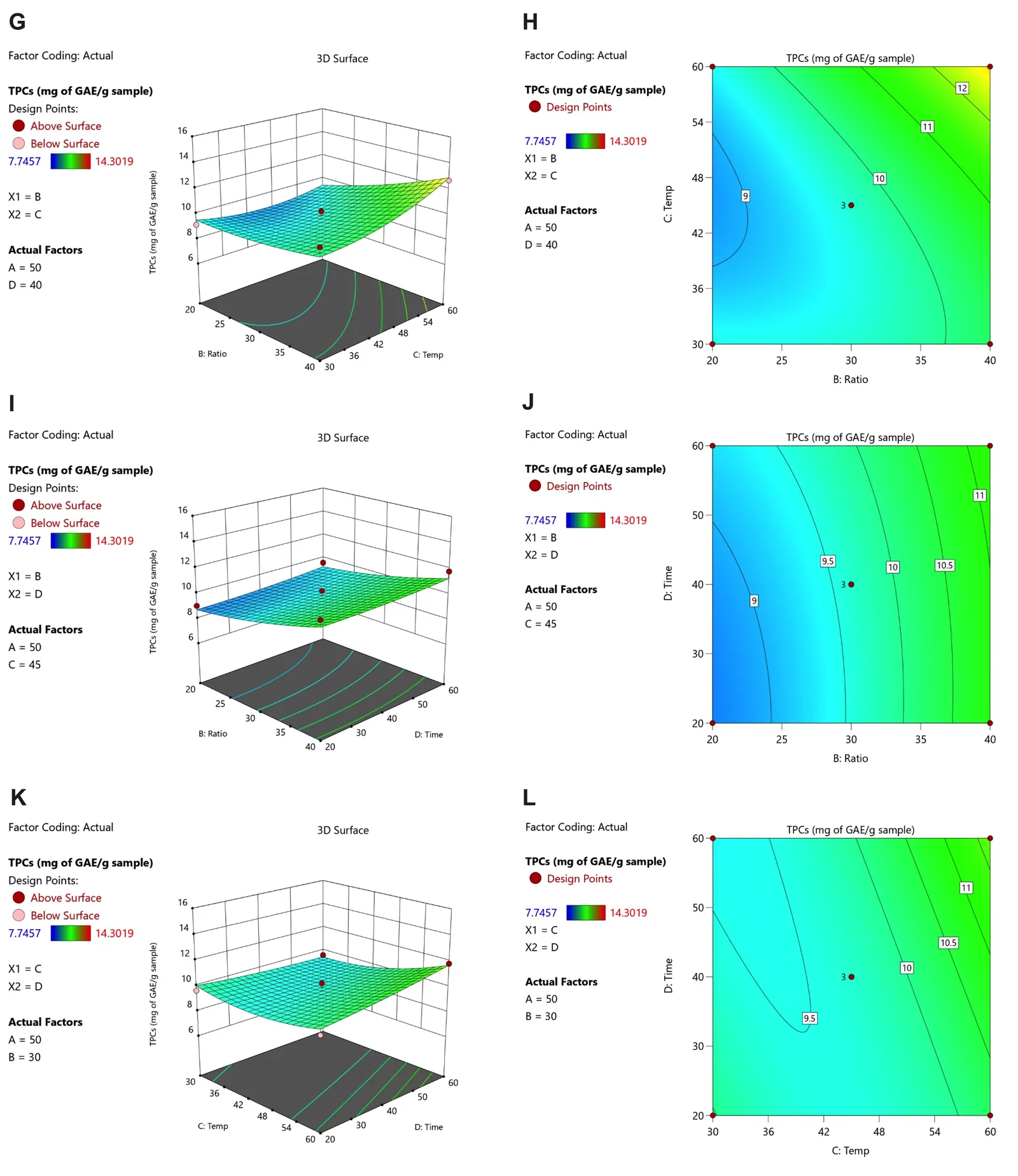
Contour plots (**A**,**C**,**E**,**G**,**I**,**K**) and response surface plots (**B**,**D**,**F**,**H**,**J**,**L**) of the total phenolic contents (TPCs) affected by ethanol concentration (% *v*/*v*), ratio, temperature, and extraction time.

**Figure 2:**
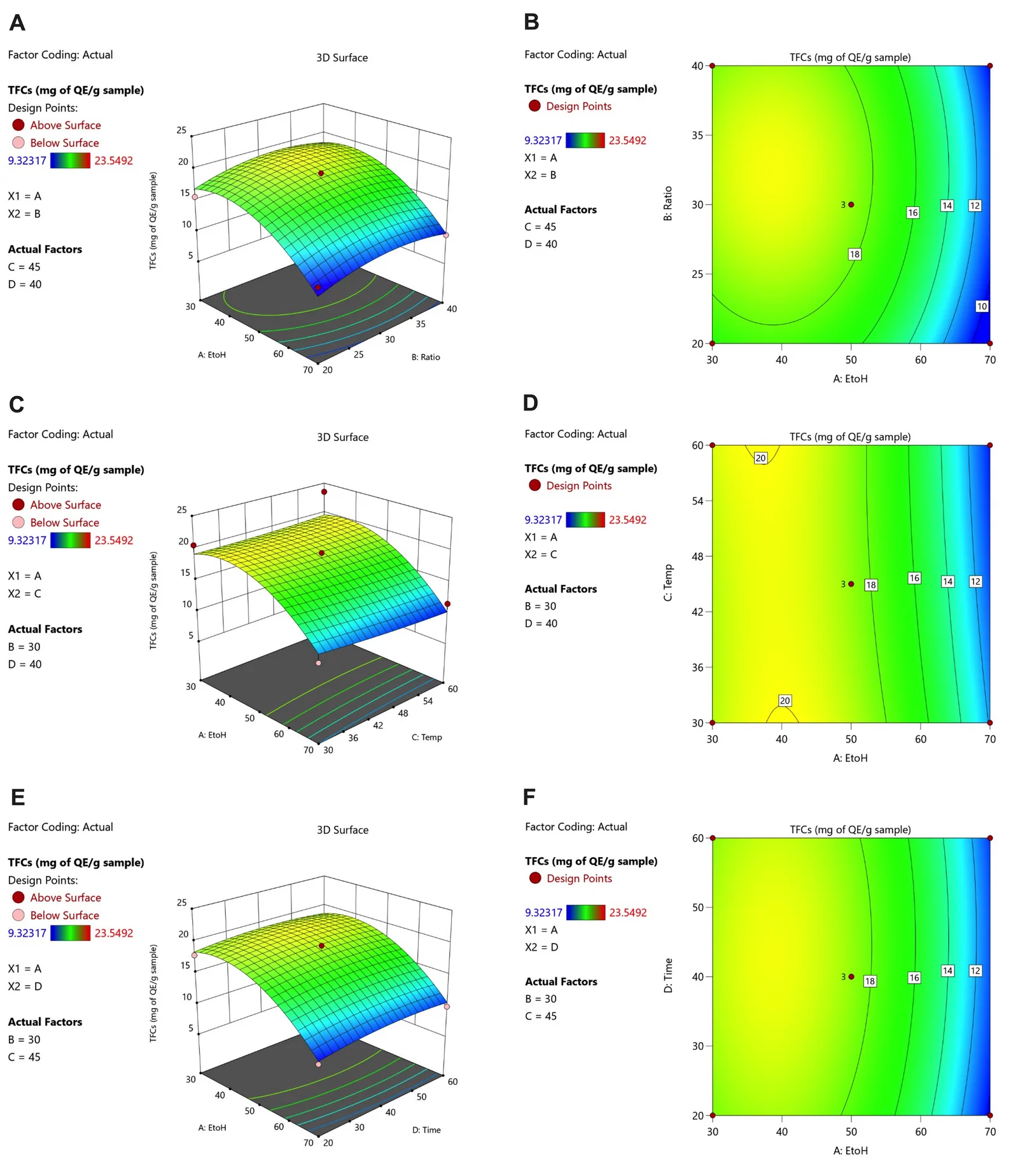

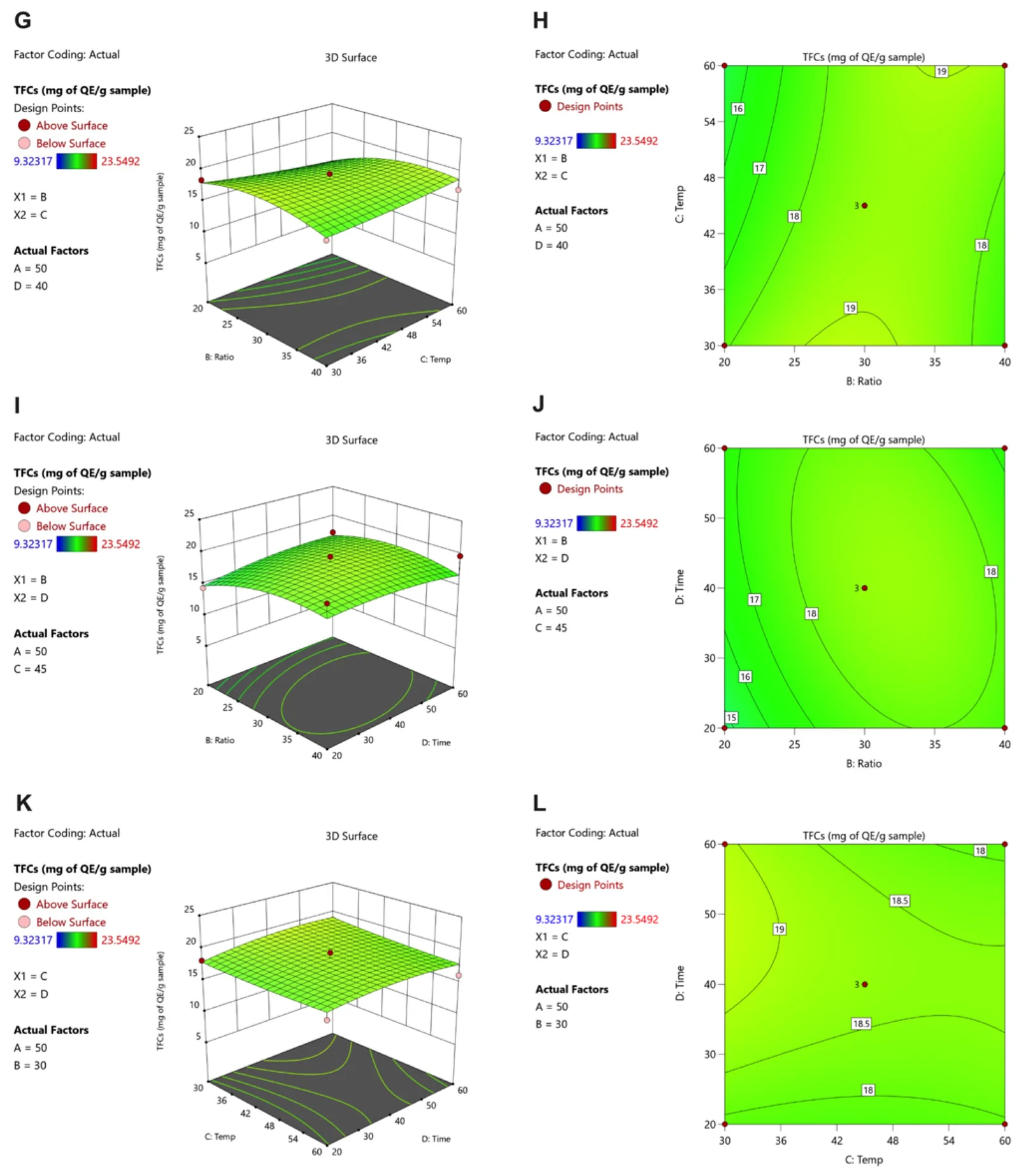
Contour plots (**A**,**C**,**E**,**G**,**I**,**K**) and response surface plots (**B**,**D**,**F**,**H**,**J**,**L**) of the total flavonoid contents (TFCs) affected by ethanol concentration (% *v*/*v*), ratio, temperature, and extraction time.

Contour and three-dimensional response surface plots (Figure 1 and Figure 2) illustrate the interactive effects of ethanol concentration, solid-to-solvent ratio, temperature, and time on TPCs and TFCs, giving visual support to the fitted models.

#### 2.3.2. Analysis of Variance (ANOVA) and Model Validation

**Figure 3:**
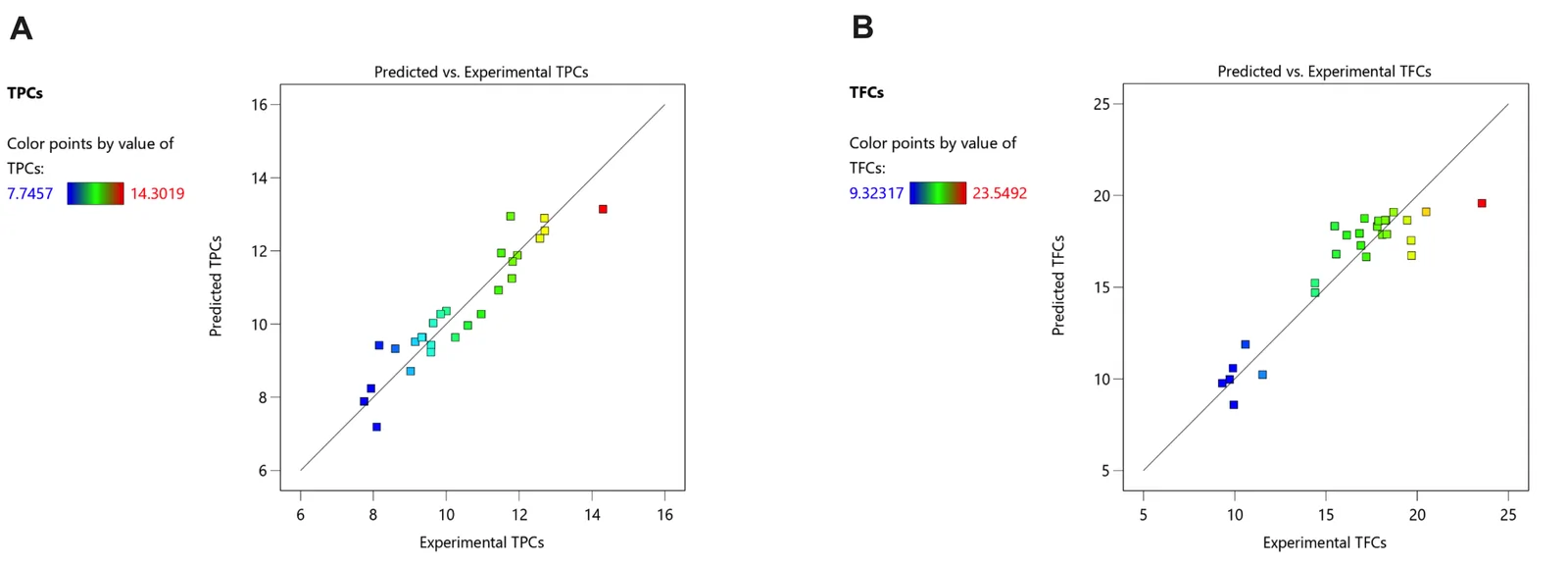
Correlation plot between the predicted and experimental values of the TPCs (**A**) and TFCs (**B**). The regression equations and coefficients of determination ($R^{2}$) for the fitted models are provided in Section 2.3.1 and Table 6, respectively.

The ANOVA data confirmed that both the TPC and TFC models were statistically significant (TPCs: *p* = 0.0013; TFCs: *p* = 0.0054), with non-significant lack-of-fit terms (TPCs: *p* = 0.2739; TFCs: *p* = 0.0804), indicating adequate model fit within the experimental domain (Table 6). This also indicates that the fitted equations for the TPCs and TFCs mentioned previously can be used for prediction. For TPCs, ethanol concentration (X_1_; *p* < 0.0001), solid-to-solvent ratio (X_2_; *p* = 0.0011), and extraction temperature (X_3_; *p* = 0.0261) were significant linear factors, whereas extraction time (X_4_) and interaction/second-order terms had lesser influence. For TFCs, ethanol concentration (X_1_; *p* < 0.0001) and its quadratic term X_1_^2^ (*p* = 0.0022) were the significant contributors, highlighting the pivotal role of solvent strength in flavonoid recovery. The coefficient of determination (R^2^) values were 0.8831 (TPCs) and 0.8453 (TFCs), with adjusted R^2^ values of 0.7467 and 0.6649, respectively. These values indicate that the models explain a substantial proportion of the variability in the responses, while the adjusted R^2^ values reflect acceptable predictive performance after accounting for the number of terms in the models. The correlation plots between the predicted and experimental values (Figure 3) further support model reliability, showing close clustering around the line of identity for both TPCs and TFCs.

#### 2.3.3. Optimal Conditions and Validation

In this study, numerical optimization was performed to identify the optimal extraction condition for achieving desirable levels of both TPCs and TFCs in the *W. globosa* extract under practical constraints, rather than the maximum of either response alone. The desirability-based optimization identified 32% ethanol, a solid-to-solvent ratio of 1:40 g/mL, extraction temperature of 60 °C, and extraction time of 28 min as the optimal conditions, with predicted TPC and TFC values of 14.30 mg GAE/g DW and 20.31 mg QE/g DW, respectively. This condition corresponded to the highest overall desirability (D = 0.879) among several candidate solutions generated by the simultaneous numerical optimization of both TPCs and TFCs. Experimental validation under these optimal conditions yielded a TPC of 15.17 ± 0.27 mg GAE/g DW and a TFC of 22.34 ± 1.71 mg QE/g DW, closely matching and slightly exceeding the predicted values. The small deviations between predicted and observed responses confirm excellent model validity and demonstrate that the optimized extraction protocol achieves simultaneous enhancement of both TPCs and TFCs in *W. globosa* extracts.

### 2.4. Antioxidant Capacity of the Optimized W. globosa Extract (WGE)

Under the optimized extraction conditions (32% ethanol, 1:40 g/mL, 60 °C, 28 min), the optimized *W. globosa* extract (WGE) exhibited a total tannin content (TTC) of 21.13 ± 0.22 mg TAE/g DW, alongside a TPC of 15.17 ± 0.27 mg GAE/g DW and a TFC of 22.34 ± 1.71 mg QE/g DW. Antioxidant activities determined by three complementary assays were: ORAC 4374.33 ± 395.65 µmol TE/g DW, FRAP 210.64 ± 1.96 µmol TE/g DW, and DPPH 136.95 ± 4.22 µmol TE/g DW (Table 7).

The markedly elevated ORAC value relative to FRAP and DPPH reflects the strong peroxyl radical-scavenging capacity of the optimized extract, consistent with a predominant hydrogen atom transfer (HAT) mechanism. This profile is in line with the flavone- and phenolic-rich composition of *W. globosa* under controlled cultivation and optimized extraction, which favors compounds such as luteolin and apigenin known to contribute robust HAT-based antioxidant activity [11].

### 2.5. Enzyme Inhibition and Synergistic Effects

#### 2.5.1. Multitarget Enzyme Inhibition of Optimized *W. globosa* Extract (WGE)

At a fixed concentration of 2 mg/mL, the optimized WGE showed differential inhibitory activity across the tested enzymes (Table 8). AChE inhibition was 13.88 ± 1.38%, BChE 15.61 ± 1.28%, and BACE-1 56.94 ± 3.05%, respectively. WGE also showed less and non-detectable enzyme inhibition against other chronic diseases (Supplementary Table S1). The markedly higher inhibition of BACE-1 relative to AChE, BChE, and metabolic enzymes indicates a clear selectivity toward the amyloidogenic pathway, suggesting that WGE preferentially targets beta-secretase activity rather than cholinesterases.

Dose–response analysis yielded IC_50_ values of 7.42 ± 0.16 mg/mL for AChE, 6.55 ± 0.24 mg/mL for BChE, and 1.59 ± 0.11 mg/mL for BACE-1 (Table 8). The notably lower IC_50_ for BACE-1—approximately 4.5-fold lower than for AChE and 4.1-fold lower than for BChE—confirms that BACE-1 is the primary enzymatic target of the extract’s bioactive constituents, consistent with the presence of flavonoids and phenolic acids previously reported to inhibit BACE-1 and modulate amyloidogenic processing.

#### 2.5.2. Combined Inhibition with Donepezil

Donepezil has served as a principal pharmacotherapy for AD for decades, functioning as a reversible, non-competitive inhibitor of AChE, and to a lesser extent, butyrylcholinesterase (BChE), thereby enhancing cholinergic neurotransmission by preventing acetylcholine degradation [24,25]. Beyond its established cholinesterase-inhibitory activity, donepezil may influence amyloid precursor protein processing and has been associated with reduced BACE1 expression in experimental studies, although direct BACE1 inhibition has not been firmly established [44]. While clinically beneficial in the short- to medium-term, donepezil therapy is associated with dose-dependent cholinergic adverse effects and demonstrates diminishing efficacy over time, underscoring the clinical necessity for complementary therapeutic approaches [26,27,28].

To assess the combined effects of WGE and donepezil on key neurodegenerative enzymes, single-dose combination experiments were performed for AChE, BChE, and BACE-1. Donepezil alone achieved inhibitory activities of 48.10 ± 1.23% (AChE), 50.10 ± 1.09% (BChE), and 52.11 ± 1.17% (BACE-1), while WF alone at the tested concentrations (7300, 6500, and 1600 µg/mL for AChE, BChE, and BACE-1, respectively) yielded 49.35 ± 1.74%, 49.35 ± 1.74%, and 50.72 ± 0.67%, respectively (Table 9).

Theoretical additive values were calculated using the standard additivity equation, giving 48.725% for AChE, 49.725% for BChE, and 51.415% for BACE-1. At the tested combination doses, WF plus donepezil produced experimental inhibitions of 60.32 ± 0.56% (AChE), 77.02 ± 0.19% (BChE), and 82.16 ± 1.88% (BACE-1), all substantially exceeding the corresponding theoretical additive values. These results demonstrate greater-than-additive (supra-additive) inhibition for all three enzymes at this single dose combination, indicating that WGE can enhance the inhibitory effects of donepezil on both cholinesterase and BACE-1.

Because only one combination concentration was examined for each enzyme, these findings are best interpreted as evidence of supra-additive effects at the tested dose, rather than definitive proof of dose-range synergism. Formal synergy analysis across multiple dose combinations and fractional effect levels would be required to conclusively establish synergistic interactions in the Chou–Talalay sense.

### 2.6. High-Performance Liquid Chromatography (HPLC) with Tandem Quadrupole Mass Spectrometer (HPLC-qTOF-MS)

Untargeted high-resolution HPLC-qTOF metabolomics provided a more comprehensive picture of the chemical space in the optimized extract, revealing both polar phenolics and complex lipids. In negative ionization mode, 12 compounds were annotated, dominated by pinolenic acid (RT 20.89 min; molecular mass 277.2197; library score 90.8; 64.82% of the total annotated area), followed by 1-palmitoyl-2-hydroxy-sn-glycero-3-phospho-(1′-rac-glycerol) (26.40%), caffeic acid (3.55%), luteolin (2.70%), ellagic acid (1.29%), and p-coumaric acid (0.37%) (Table 10). The presence of pinolenic acid and lysophospholipids suggests that the optimized extraction also enriches nutritionally relevant fatty acids and phospholipids, which may contribute to membrane-related and anti-inflammatory effects.

In positive ionization mode, 38 compounds were annotated, including the prominent flavone C-glycosides isovitexin (26.35%) and schaftoside (12.93%), as well as phosphatidylcholines (e.g., 1-palmitoyl-sn-glycero-3-phosphocholine, 10.91%), monolinolenin (6.09%), phenylalanine (3.47%), adenosine (2.57%), α-tocopherol (1.85%), campesterol (0.28%), luteolin (0.58%), and L-tryptophan (1.64%) (Table 11).

The consistent detection of luteolin, isovitexin, schaftoside, caffeic acid, p-coumaric acid, and alpha-tocopherol across modes supports a phytochemical profile enriched in flavones, flavone C-glycosides, phenolic acids, and lipid-soluble antioxidants, all of which have documented antioxidant and neuroprotective properties [21,27,29,45,46,47]. In particular, isovitexin and schaftoside are known constituents of *W. globosa* and other duckweeds, and their abundance here aligns with the strong ORAC values and the observed BACE-1 inhibitory selectivity.

### 2.7. Targeted LC-MS/MS Analysis

Targeted LC-MS/MS analysis revealed distinct phenolic profiles under acid and non-acid hydrolysis conditions (Table 12 and Figure S1), reflecting differences between aglycone and glycosidically bound forms. Under non-acid hydrolysis of the native extract, luteolin levels increased markedly to 12.18 ± 0.17 µg/mL, accompanied by caffeic acid at 1.69 ± 0.01 µg/mL, while apigenin and naringenin were not detected at comparable levels. Under acid hydrolysis, the major flavonoid aglycones detected were luteolin (1.98 ± 0.01 µg/mL), apigenin (0.77 ± 0.05 µg/mL), and naringenin (0.32 ± 0.01 µg/mL), indicating that these compounds are present in conjugated forms that can be released by acid treatment. This pattern suggests that luteolin predominantly exists in the glycosidically bound form in *W. globosa*, which is efficiently liberated under the optimized extraction conditions, whereas apigenin and naringenin are present mainly as minor aglycones or less abundant conjugates. The co-occurrence of caffeic acid supports a phenolic acid–flavone enrichment profile consistent with the observed antioxidant and BACE-1 inhibitory activities.

### 2.8. Molecular Docking

**Figure 4:**
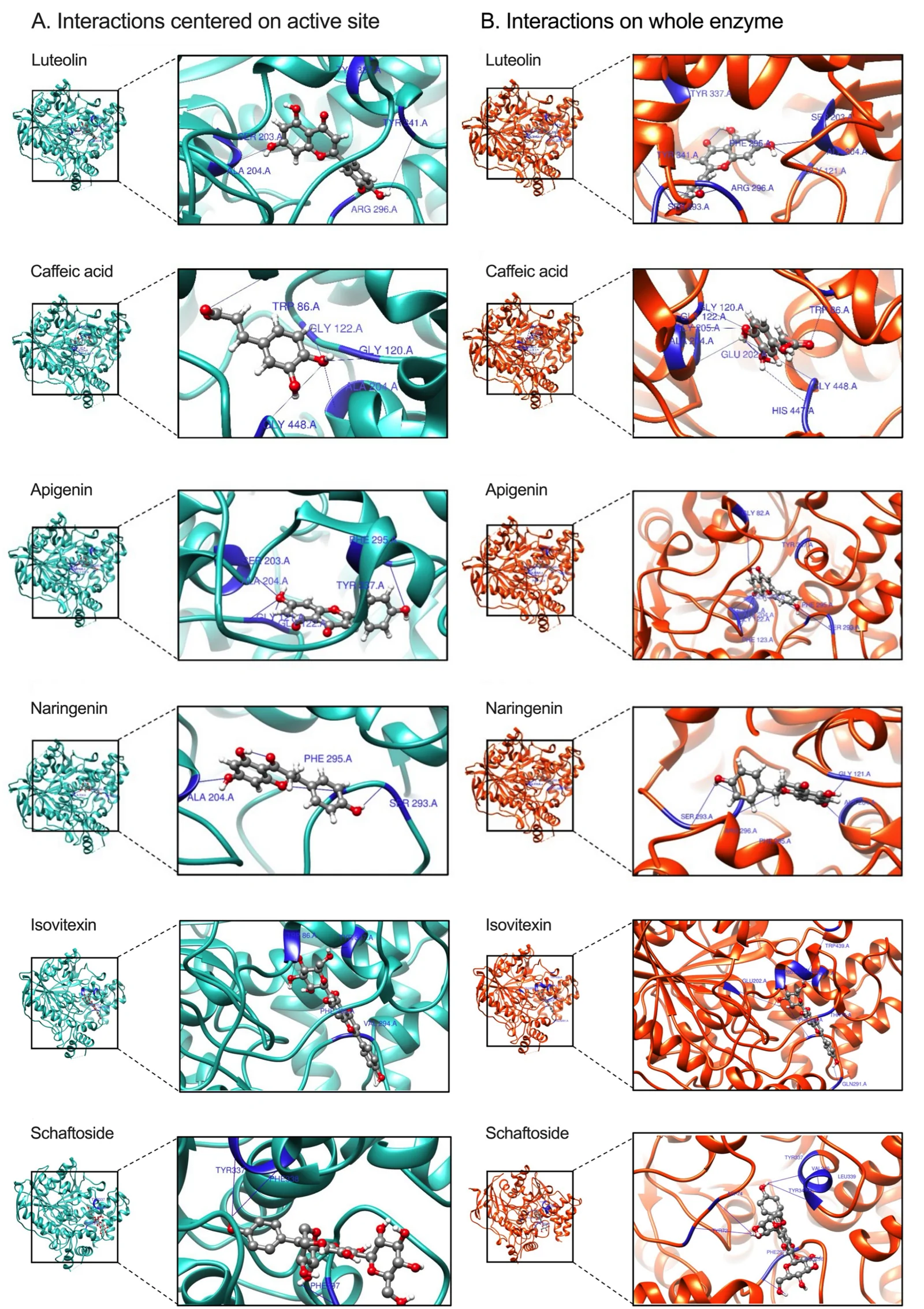
The 3D structural prediction of each chemical compound (blue stick conformation) in the catalytic pocket of AChE (light sea green ribbon structure, PDB: 7E3H) on interactions centered on the active site (**A**). The 3D structural prediction of each chemical compound (blue stick conformation) in the catalytic pocket of acetylcholinesterase (orange ribbon structure, PDB: 7E3H) on interactions centered on the macromolecule (**B**).

**Figure 5:**
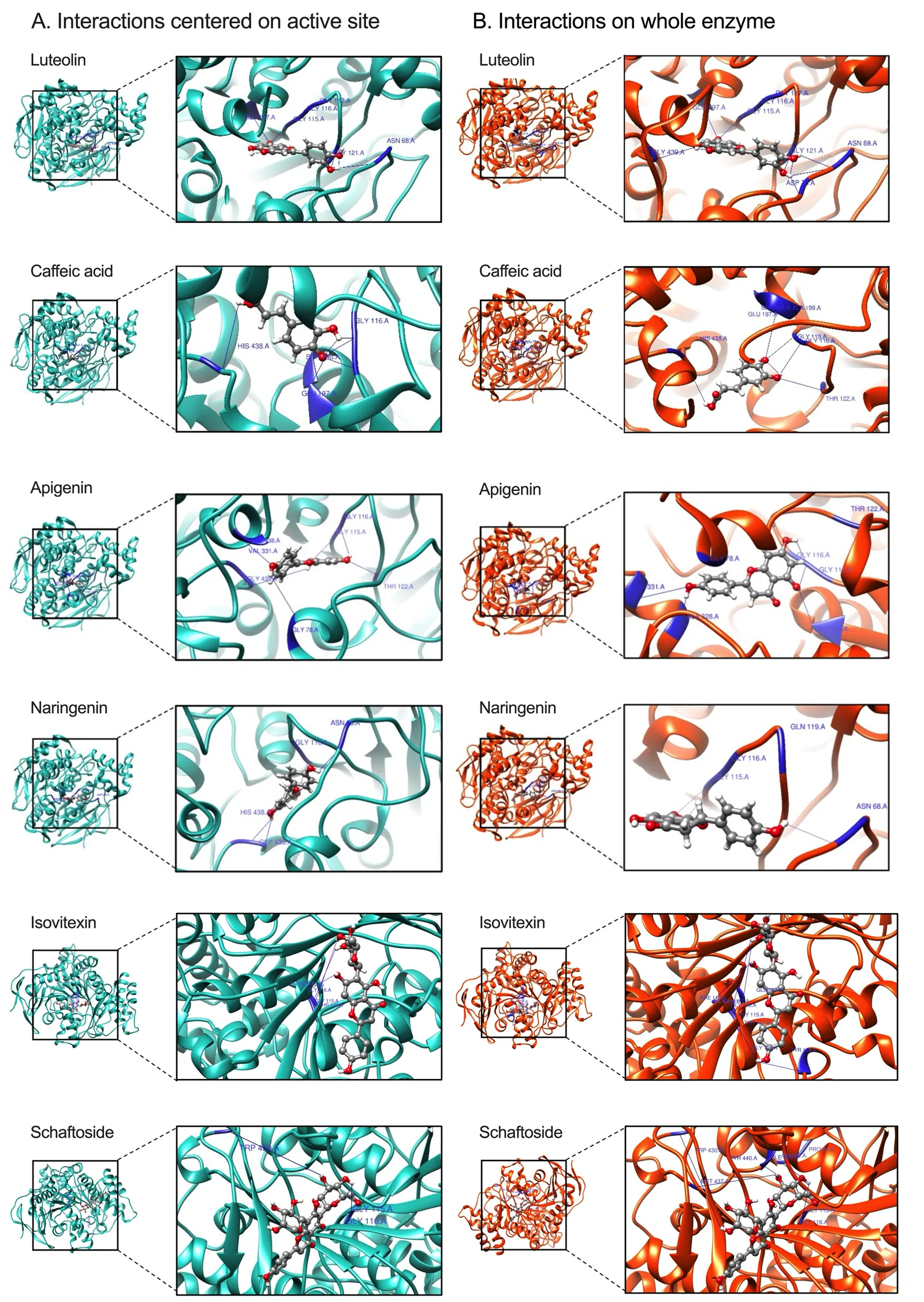
The 3D structural prediction of each chemical compound (blue stick conformation) in the catalytic pocket of BChE (light sea green ribbon structure, PDB: 4TPK) on interactions centered on the active site and (**A**) the 3D structural prediction of each chemical compound (blue stick conformation) in the catalytic pocket of butyrylcholinesterase (orange ribbon structure, PDB: 4TPK) on interactions centered on the macromolecule (**B**).

**Figure 6:**
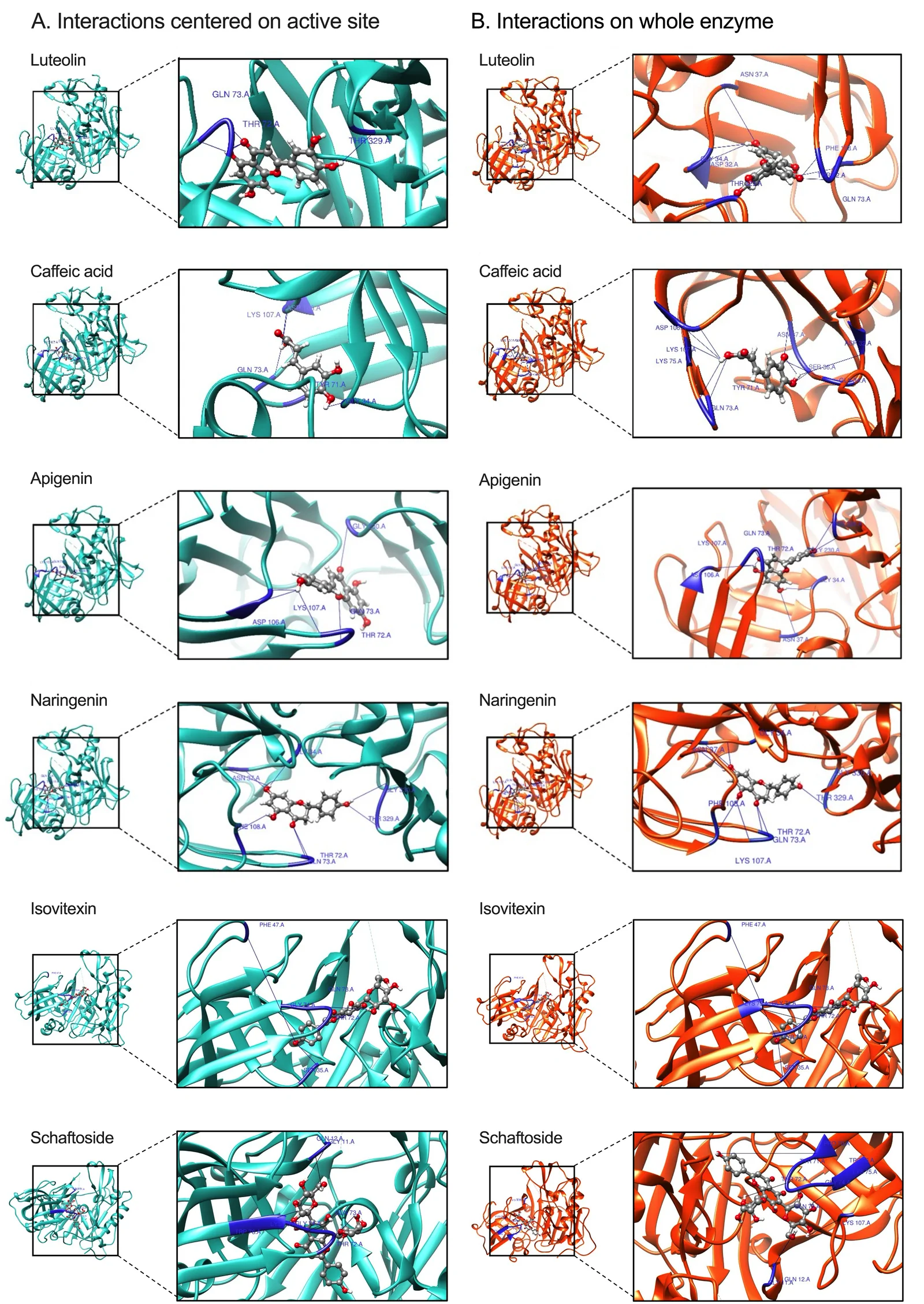
The 3D structural prediction of each chemical compound (blue stick conformation) in the catalytic pocket of BACE-1 (light sea green ribbon structure, PDB: 6EQM) on interactions centered on the active site (**A**). The 3D structural prediction of each chemical compound (blue stick conformation) in the catalytic pocket of beta-secretase (orange ribbon structure, PDB: 6EQM) on interactions centered on the macromolecule (**B**).

Molecular docking was performed to explore the interactions between major phenolic compounds identified in the optimized WGE extract (luteolin, apigenin, naringenin, and caffeic acid) and human acetylcholinesterase (AChE; PDB: 7E3H), butyrylcholinesterase (BChE; PDB: 4TPK), and beta-secretase 1 (BACE-1; PDB: 6EQM), as shown in Figure 4, Figure 5 and Figure 6. Two complementary approaches were used for docking for each enzyme: docking on the active site and blind docking on the whole enzyme surface. This dual approach was chosen because there is no single mode of binding for enzyme inhibitors. Competitive inhibitors bind in the active site, whereas noncompetitive, uncompetitive, and allosteric inhibitors bind at sites that are topographically different from the catalytic pocket [48]. Active-site-centered docking probes competitive binding in the catalytic pocket. Whole-enzyme docking explores the entire molecular surface without any positional bias and may identify energetically favorable binding at peripheral or allosteric regions that would be missed by a predefined active-site grid [49].

#### 2.8.1. Docking to AChE (PDB: 7E3H)

For AChE, luteolin exhibited the highest binding affinity at the active site (estimated free binding energy: −10.243 kcal/mol), forming hydrogen bonds with SER203, ALA204, ARG296, TYR337, and TYR341 (Table 13). Naringenin showed comparable affinity (−10.059 kcal/mol), followed by apigenin (−9.883 kcal/mol) and caffeic acid (−7.503 kcal/mol).

Docking centered on the macromolecule produced similar affinities (e.g., luteolin −10.213 kcal/mol), with additional contacts involving GLY121, SER293, PHE295, and ARG296, suggesting that luteolin and related flavones can effectively occupy the catalytic gorge and interact with the catalytic triad and peripheral anionic site. The two most abundant flavone C-glycosides of the extract, isovitexin and schaftoside, were also docked with AChE. Like the flavone aglycones, isovitexin was strongly bound at the active site (−10.137 kcal/mol; 2 hydrogen bonds; TRP86, VAL294, PHE295, TYR337) and showed similar affinity in the whole-macromolecule docking (−9.820 kcal/mol; 3 hydrogen bonds; THR75, GLU84, TRP86, GLU202, GLN291, VAL294, PHE295, TYR337, TRP439). Schaftoside showed a weaker interaction at the active site (−7.169 kcal/mol; 1 hydrogen bond; PHE297, TYR337, PHE338), but a better interaction when the whole macro-molecule was explored (−8.630 kcal/mol; 3 hydrogen bonds; TYR72, ASP74, ARG296, PHE297, TYR337, PHE338, LEU339, VAL340, TYR341).

#### 2.8.2. Docking to BChE (PDB: 4TPK)

For BChE active site docking, luteolin showed the highest docking score (−9.462 kcal/mol) followed by isovitexin (−9.319 kcal/mol), apigenin (−9.164 kcal/mol), naringenin (−9.062 kcal/mol), schaftoside (−6.952 kcal/mol), and caffeic acid (−6.532 kcal/mol) (Table 14). Luteolin formed hydrogen bonds with Asn68, Gly115-117, Gly121, and Glu197, thus placing it in the catalytic region and permitting interactions with residues involved in substrate recognition. Apigenin and naringenin showed similar docking scores, and they interacted with Gly78, Gly115–116, Thr122, His438, and Gly439. Conversely, caffeic acid showed lower docking score and fewer stabilizing contacts. The interactions of isovitexin involved one hydrogen bond with Gly115, Gly116, Gly117, Gln119, and Thr120, whereas schaftoside formed one hydrogen bond and interacted with Gly115, Gly116, and Trp430. Overall, the active site docking results indicate that several flavonoids identified in *W. globosa*, mainly luteolin and isovitexin, could favorably interact with the catalytic region of BChE, possibly contributing to the moderate inhibition of BChE observed in vitro. The docking scores of isovitexin and schaftoside were −7.540 and −6.233 kcal/mol by whole-enzyme docking, which were less favorable than the corresponding active-site docking scores.

#### 2.8.3. Docking to BACE-1 (PDB: 6EQM)

Luteolin showed the most favorable docking score (−8.867 kcal/mol), followed by naringenin (−8.760 kcal/mol), apigenin (−8.664 kcal/mol), caffeic acid (−7.703 kcal/mol), isovitexin (−7.546 kcal/mol), and schaftoside (−7.210 kcal/mol) for BACE-1 active-site docking (Table 15). Hydrogen bonds were formed by naringenin with Gly34, Asn37, Thr72, Gln73, Phe108, Thr329, and Gly330. Luteolin and apigenin showed similar docking scores and interacted with Thr72, Gln73, Asp106, Lys107, and Gly230. Although caffeic acid had a poorer score, it formed several hydrogen bonds with Gly34, Tyr71, Gln73, Asp106, and Lys107. Isovitexin formed one hydrogen bond and contacted Ser35, Phe47, Thr72, Gln73, and Gly74 while schaftoside formed two hydrogen bonds and contacted Gly11, Gln12, Thr72, Gln73, Gly74, Lys75, and Trp76. Both C-glycosides showed interactions with Thr72 and Gln73, but their docking scores were less favorable than the scores of the flavone aglycones, naringenin and caffeic acid.

Whole enzyme docking yielded generally similar scores and identified additional contacts with the catalytic residue Asp32, as well as Gly34, Asn37, Phe108, and Thr329. The whole-enzyme docking scores for isovitexin and schaftoside were −6.660 and −6.100 kcal/mol, respectively, which were less favorable than their respective active-site docking scores. These docking patterns collectively suggest that some flavonoids and phenolic acids of W. globosa can be accommodated in the BACE-1 active pocket and interact with residues involved in catalysis and substrate recognition. These results provide a possible structural rationale for the relatively potent inhibition of BACE-1 observed in vitro, although docking results alone cannot prove the formation or stability of nonproductive enzyme–ligand complexes.

#### 2.8.4. Molecular Dynamics (MD) Simulation

Since molecular docking considers ligand binding as a static, rigid snapshot and does not take into account the flexibility of proteins and ligands or explicit solvent effects, the docked complexes were studied by means of MD simulation to evaluate the dynamic stability of the predicted binding poses during the simulation time [50,51]. The trajectory of each of the six ligands (luteolin, caffeic acid, apigenin, naringenin, isovitexin and schaftoside) complexed with AChE, BChE, and BACE-1 was analyzed using the RMSD to monitor the overall stability of the complex and the RMSF to assess the per-residue flexibility of the enzymes.

The RMSD of all six ligands for the AChE complexes (Figure S2) increased during the first ~100–200 ps and then reached equilibrium, fluctuating in a range of about 0.15–0.25 nm for the rest of the trajectory, indicating stable complexes, with luteolin and isovitexin showing the lowest and most stable deviations. Also, the BChE complexes (Figure S3) were stabilized, reaching plateaus at higher RMSD values of ~0.35–0.50 nm, with the largest deviations observed for caffeic acid and apigenin, and isovitexin being the most stable. In contrast, the BACE-1 complexes (Figure S4) exhibited higher RMSD values (~2–6 nm) that increased over time throughout the simulation for luteolin, apigenin, naringenin, and isovitexin, with stepwise increases observed for naringenin and isovitexin, while caffeic acid and schaftoside were relatively more stable (~2–3 nm).

RMSF profiles per residue were consistent with RMSD behavior. For AChE (Figure S5) and BChE (Figure S6), the enzymes showed a low degree of total flexibility with many amino acid residues fluctuating within the range of ~0.05–0.10 nm and only localized peaks, consistent with stable, well-maintained ligand-bound conformations. The baseline RMSF for BACE-1 was also low (~0.05–0.10 nm) (Figure S7) but with notable peaks (~0.3–0.4 nm) in particular loop regions (~280–360 residues), suggesting enhanced local flexibility of the BACE-1 structure throughout the simulation.

### 2.9. In Vivo Validation in D. melanogaster Expressing Human APP and BACE-1

#### 2.9.1. Climbing Score (Negative Geotaxis Assay)

**Figure 7:**
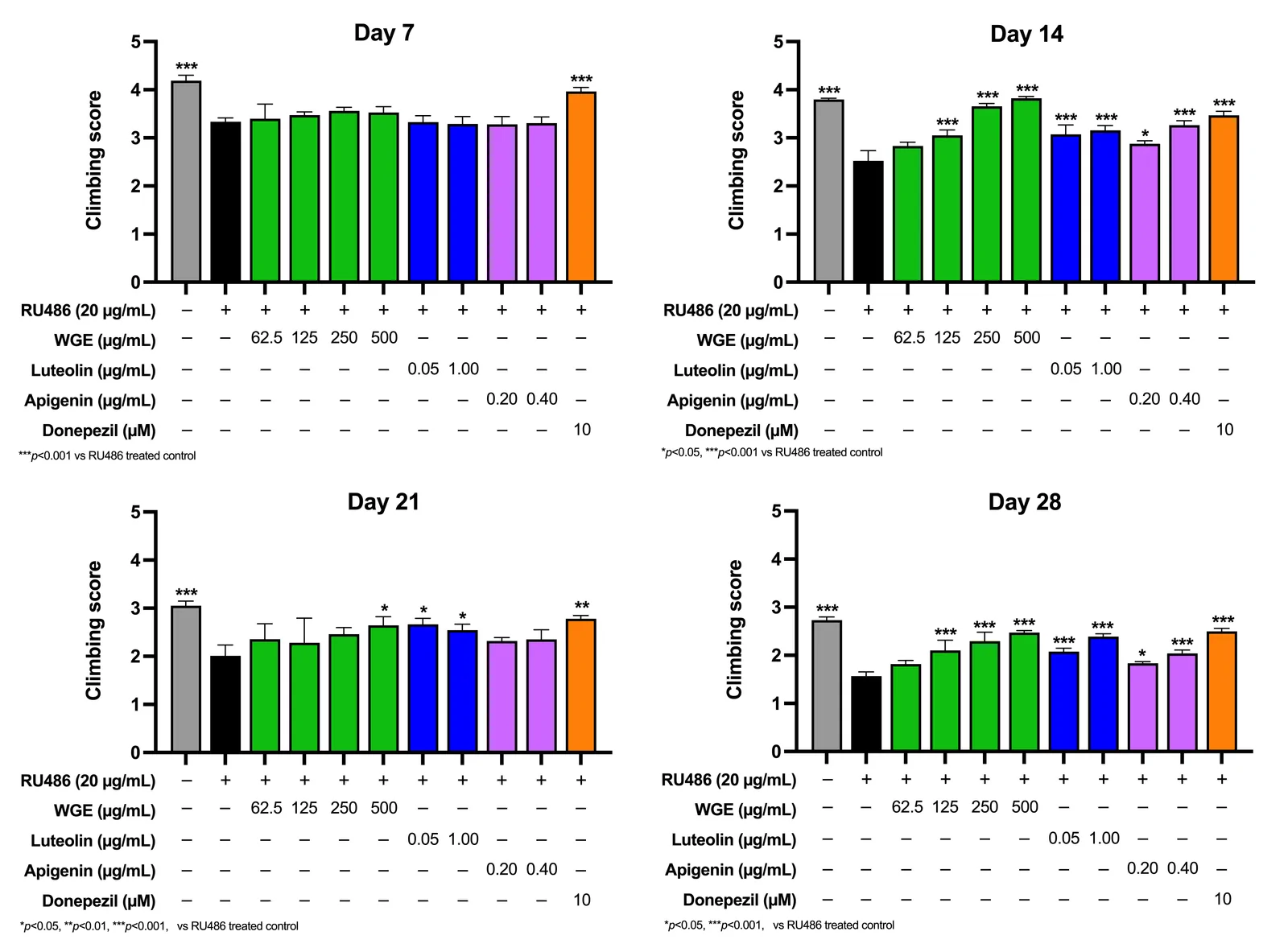
Effect of RU486, WGE, luteolin, apigenin, donepezil, and their combinations on locomotor function (climbing score) of adult flies at 7, 14, 21, and 28 days. Values are mean ± SD of three independent assays. One-way ANOVA followed by Tukey’s test was used to determine statistical differences between groups, * p < 0.05, ** p < 0.01, *** *p* < 0.001 versus RU486-treated alone.

RU486-fed flies (AD model group), which induced the expression of both human APP and BACE-1, thereby representing an AD model of the amyloidogenic pathway, exhibited a progressive decline in climbing performance at 7, 14, 21, and 28 days compared with the untreated control group (no AD control). At day seven, WGE did not significantly improve climbing relative to RU486 alone, whereas donepezil (10 µM) markedly restored locomotor function. From day 14 onward, WGE produced significant, dose-dependent improvements in climbing scores, with higher doses (125–500 µg/mL) showing clear protection at days 14, 21, and 28 (*p* < 0.05–0.001, Figure 7). At corresponding time points, the protective effect of WGE at its higher doses was comparable to, and in some cases approached, that of the donepezil positive control, indicating that the extract confers meaningful in vivo neuroprotective benefits on locomotor function. Treatment with individual flavonoids (luteolin and apigenin) also significantly improved climbing scores at later time points, and their effects, alone or combined with donepezil, further support the contribution of these constituents to the observed motor protection relative to RU486-only flies.

#### 2.9.2. Brain BACE-1 Activity

**Figure 8:**
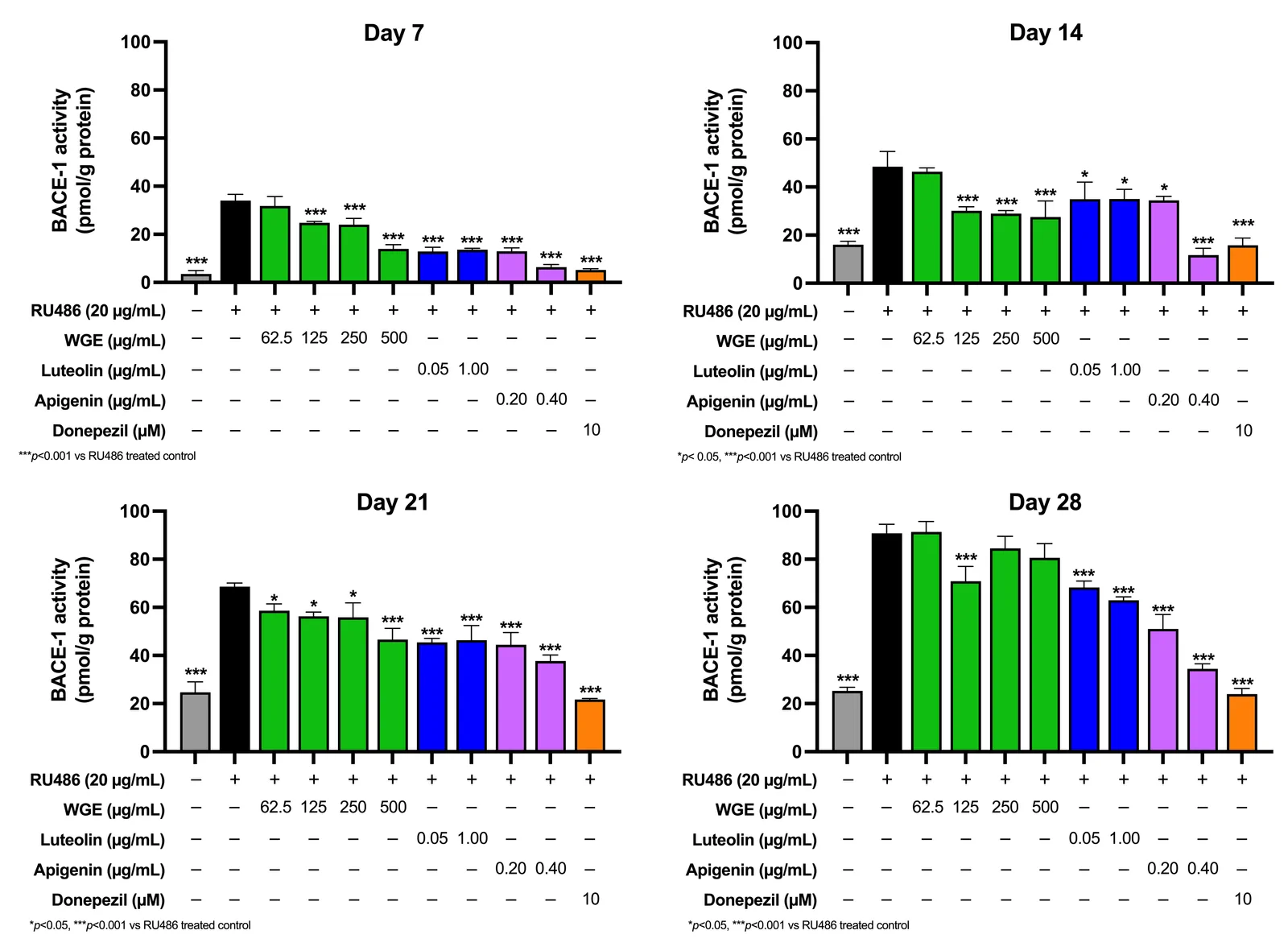
Effect of RU486, WGE, luteolin, apigenin, donepezil, and their combinations on brain BACE-1 activity in adult flies at 7, 14, 21, and 28 days. Values are mean ± SD of three independent assays. One-way ANOVA followed by Tukey’s test was used to determine the differences between indicated groups. *, *p* < 0.05 and ***, *p* < 0.001 compared to RU486-treated alone.

RU486 administration significantly increased brain BACE-1 activity in *Drosophila* at all examined time points (7, 14, 21, and 28 days) compared with the untreated control group (*p* < 0.001), confirming effective induction of an amyloidogenic AD-like phenotype. At day seven, WGE at 125–500 µg/mL, luteolin at both 0.05 and 1.00 µg/mL, apigenin at 0.40 µg/mL, and donepezil 10 µM all significantly reduced BACE-1 activity relative to the RU486-treated flies (* *p* < 0.001), whereas WGE 62.5 µg/mL showed a non-significant trend toward reduction (Figure 8).

By day 14, WGE at 125–500 µg/mL again significantly lowered BACE-1 activity (*p* < 0.001), with WGE 62.5 µg/mL and luteolin 1.00 µg/mL showing moderate but significant reductions (*p* < 0.05), and apigenin 0.40 µg/mL and donepezil 10 µM producing robust decreases (*p* < 0.001). At day 21, the lowest WGE dose (62.5 µg/mL) no longer differed significantly from RU486, whereas higher doses (125–500 µg/mL), luteolin 1.00 µg/mL, apigenin 0.40 µg/mL, and donepezil 10 µM remained significantly protective (*p* < 0.05–*p* < 0.001). By day 28, WGE at 250–500 µg/mL, luteolin 1.00 µg/mL, apigenin 0.40 µg/mL, and donepezil 10 µM continued to significantly attenuate BACE-1 activity compared with RU486 alone (* *p* < 0.001), whereas the lowest WGE and flavonoid doses showed partial but less pronounced effects (Figure 8).

Taken together, these results demonstrate that *W. globosa* extract, as well as its major flavonoids luteolin and apigenin, can significantly suppress RU486-induced elevation of brain BACE-1 activity in a dose- and time-dependent manner, with higher doses approaching the inhibitory effects of donepezil. This in vivo BACE-1 modulation is consistent with the strong in vitro BACE-1 inhibition and supports the potential of *W. globosa* as a nutraceutical candidate targeting amyloidogenic processing.

## 3. Discussion

The efficiency of bioactive compound recovery from plant matrices is strongly influenced by solvent polarity, solid-to-solvent ratio, extraction temperature, and extraction duration [52,53,54]. In the preliminary solvent-screening experiment, 70% (*v*/*v*) aqueous ethanol yielded higher TPC and TFC values than the non-polar solvents and pure water tested. This observation is consistent with the ability of hydroethanolic solvents to extract phenolic compounds with diverse polarities [53,54,55]. However, the preliminary solvent comparison should not be interpreted as defining the final optimal extraction condition because extraction performance is also influenced by the interaction of ethanol concentration with solid-to-solvent ratio, temperature, and extraction time. The two-phase evaluation further showed that the aqueous extract exhibited lower measured TPC after evaporation and reconstitution, declining from 14.73 to 6.97 mg GAE/g DW. In contrast, the ethanol extract showed a small increase in measured TPC after evaporation and reconcentration, from 15.68 to 16.38 mg GAE/g DW. These findings indicate that the measured phenolic content was affected by post-extraction processing. However, the present data do not establish whether these changes resulted from thermal degradation, concentration effects, incomplete recovery during reconstitution, or other matrix-related factors. Therefore, the mechanistic basis for the observed differences requires further investigation.

Following simultaneous optimization of the extraction variables using the BBD–RSM approach, the final optimized conditions were 32% (*v*/*v*) aqueous ethanol, a solid-to-solvent ratio of 1:40 g/mL, an extraction temperature of 60 °C, and an extraction duration of 28 min. The second-order polynomial models showed $R^{2}$ values of 0.8831 for TPC and 0.8453 for TFC, with non-significant lack-of-fit tests (p=0.2739 and p=0.0804, respectively). Experimental validation under the predicted optimum conditions produced prediction errors of 6.1% for TPC and 10.0% for TFC, supporting the practical utility of the models for the studied extraction system. The extract prepared under these optimized conditions was used for all subsequent chemical and biological experiments [38,56].

The optimized WGE demonstrated high antioxidant capacity across three mechanistically complementary assays. The markedly elevated ORAC value relative to FRAP and DPPH is consistent with a hydrogen atom transfer (HAT)-dominant antioxidant mechanism, reflecting the strong capacity of *W. globosa* flavones, particularly luteolin and apigenin, for peroxyl radical chain-breaking via rapid H-atom donation from catechol hydroxyl groups on the B-ring [45,57,58]. The co-presence of caffeic acid further broadens the antioxidant mechanism, as hydroxycinnamic acids such as caffeic and p-coumaric acids rank among the most potent HAT-active phenolic acids. The total tannin content suggests that condensed and hydrolysable tannins constitute an additional bioactive class contributing to radical scavenging, metal chelation, and protein-binding antioxidant activities [59].

A notable finding was that WGE exhibited measurable in vitro BACE-1 inhibitory activity. The IC_50_ of WGE against BACE-1 was approximately 4.5-fold lower than that against AChE and 4.1-fold lower than that against BChE, indicating greater apparent inhibitory potency toward BACE-1 than toward the cholinesterases under the assay conditions used. BACE-1 contributes to amyloidogenic APP processing, and together with γ-secretase, is involved in the generation of Aβ peptides implicated in AD pathogenesis [60,61]. The relative in vitro inhibitory activity of WGE across the enzymes evaluated suggests that BACE-1-associated pathways may warrant further investigation. However, these findings do not establish BACE-1 as the primary molecular target of WGE, demonstrate pharmacological selectivity, or confirm modulation of APP processing or Aβ production in vivo. Thus, WGE should be considered as a candidate for further preclinical evaluation in AD-relevant models rather than an established amyloidogenesis-directed dietary intervention [62,63].

The IC_50_ of WGE against BACE-1 (~1.59 mg/mL) should be interpreted with caution regarding its pharmacological relevance in vivo. Because WGE is a crude extract, this concentration represents the total extract and not the concentration of an individual BACE-1-active constituent. For example, luteolin, one of the identified flavones in WGE, has been shown to inhibit BACE-1 with an IC_50_ of about 0.5 µM [64]. Therefore, a direct comparison of the IC_50_ of the crude extract in mg/mL and the IC_50_ of purified constituents in molar concentrations is not appropriate, especially as the relative contribution of each constituent to the overall BACE-1 inhibitory activity of WGE has not been determined. Importantly, the present study does not demonstrate that a systemic or brain concentration of WGE of 1.59 mg/mL can be obtained after oral administration. Indeed, the poor oral bioavailability of many polyphenolic constituents is an important translational limitation. For example, after dietary intake of about 17 mg apigenin from parsley, reported human plasma apigenin concentrations were only 28–337 nmol/L [65]. Therefore, the in vitro IC_50_ of 1.59 mg/mL cannot be considered as a therapeutically achievable concentration in plasma or brain. Nevertheless, the in vitro finding is evidence that WGE can directly inhibit BACE-1 under the assay conditions employed and may contribute to the biological effects observed in vivo. In the present *Drosophila* experiment, dietary administration of WGE at 62.5–500 μg/mL significantly decreased brain BACE-1 activity after 28 days. However, it cannot be assumed that the concentration of WGE in the diet is the same as that in the circulation or brain, as absorption, metabolism, tissue distribution and blood–brain barrier penetration of the constituents of WGE were not determined. Thus, the in vivo results confirm biological activity after oral exposure but do not demonstrate that the in vitro IC_50_ concentration was attained in brain tissue. Therefore, the safety of systemic exposure to 1.59 mg/mL cannot be inferred from the present data. Studies of pharmacokinetics and toxicology in mammalian models are required to determine achievable plasma and brain concentrations, blood–brain barrier penetration, dose–exposure relationships, and safety margins. Thus, the present BACE-1 result should be mainly considered as mechanistic evidence of inhibitory activity rather than as evidence that the measured in vitro IC50 represents a directly achievable therapeutic concentration in vivo.

Combination experiments at a single dose level showed that co-administration of WGE and donepezil produced enzyme inhibitions substantially exceeding theoretical additive values for AChE, BChE, and BACE-1. Although these results demonstrate clear supra-additive effects at the tested dose, they should be interpreted as potential synergistic interactions rather than definitive dose-range synergism, as full Chou–Talalay analysis across multiple concentration combinations was not performed. The greatest supra-additive magnitude was observed for BACE-1 and BChE, consistent with the capacity of these enzyme active sites and surrounding pockets to accommodate both the small-molecule inhibitor and additional ligands at auxiliary binding regions [66,67,68]. This interpretation is in line with the molecular docking data, which indicate that luteolin, apigenin, and naringenin engage residues partially distinct from those involved in donepezil binding [68,69,70]. Together, these findings suggest that *W. globosa* could be explored as an adjunct to existing cholinesterase inhibitors, with the potential to enhance efficacy at lower drug doses.

Metabolomic profiling provides a plausible chemical basis for the observed bioactivities. The extract contained luteolin, apigenin, naringenin, caffeic acid, and related flavonoid and lipid constituents known to contribute to antioxidant and neuroprotective effects. Among these, luteolin appears especially important because its abundance and docking behavior are consistent with a principal role in the biological activity of the extract. The presence of pinolenic acid and other lipid components may add anti-inflammatory value, broadening the functional relevance of the extract beyond polyphenols alone. [5,8,71,72,73,74].

Molecular docking against AChE (PDB: 7E3H), BChE (PDB: 4TPK), and BACE-1 (PDB: 6EQM) produced binding energy hierarchies (luteolin > apigenin ≈ naringenin > caffeic acid) that closely paralleled the experimental enzyme inhibition potency order, strongly supporting luteolin and apigenin as principal bioactive drivers [68,69,70]. For AChE, luteolin (−10.243 kcal/mol; hydrogen bonds with SER203, ALA204, ARG296, TYR337, TYR341) and naringenin (−10.059 kcal/mol; interactions including PHE295) exhibited binding energies comparable to or stronger than many reported natural polyphenolic AChE inhibitors [68,69,70]. For BChE, luteolin (−9.462 kcal/mol) engaged residues in the acyl-binding pocket and catalytic region (ASN68, GLY115–117, GLU197, HIS438), helping to explain the differential BChE selectivity. For BACE-1, naringenin (−8.760 kcal/mol; GLY34, ASN37, THR72, GLN73, PHE108, THR329) and luteolin (−8.867 kcal/mol) both contacted flap and catalytic pocket residues important for substrate recognition and inhibition [67,68,69,70]. These in silico results provide a mechanistic framework that integrates the metabolomic profile with the multitarget enzyme inhibition data. Docking of the two major flavone C-glycosides of the extract, isovitexin and schaftoside, supports these observations. Isovitexin bound to the active sites of AChE and BChE with binding energies similar to those of the flavone aglycones (−10.137 and −9.319 kcal/mol, respectively), indicating that the most abundant C-glycoside can also occupy the cholinesterase active sites, whereas both C-glycosides bound BACE-1 more weakly than the aglycones (−7.546 and −7.210 kcal/mol). For all three targets, schaftoside exhibited weaker active-site binding than isovitexin, consistent with the greater steric bulk of its di-C-glycosidic substitution. These results suggest that flavone aglycones, especially luteolin, remain the strongest predicted binders, but the abundant C-glycosides, especially isovitexin, may also contribute to the cholinesterase-directed activity of the extract.

The present bioactive findings substantially extend the recognized nutritional profile of *W. globosa*—originally noted for exceptional protein content (up to ~45% DW), complete essential amino acid composition, and rare plant-source vitamin B_12_—into validated polyfunctional nutraceutical territory [7,9,75]. The integration of high-quality protein, dietary flavone glycosides (isovitexin, schaftoside), tocopherols (α-tocopherol), phytosterols (campesterol), polyunsaturated bioactive lipids (pinolenic acid), and essential amino acids (tryptophan, phenylalanine) within a single ingredient positions *W. globosa* as a multi-class functional food [7,9,75,76]. Encapsulation or nanoformulation strategies—such as lipid-based nanoparticles, nanoemulsions, or cyclodextrin inclusion complexes—represent promising approaches to improve the oral bioavailability of hydrophobic flavone aglycones and to enhance delivery of the anti-neuroinflammatory lipid fraction [77,78,79].

The in vivo component, employing an RU486-inducible *D. melanogaster* neurodegeneration model, provided organismal-level validation of the in vitro findings. *Drosophila* has been established as a powerful and genetically tractable model for AD and other neurodegenerative diseases, with APP/BACE-1 co-expression or Aβ production in fly neurons producing progressive Aβ accumulation, synaptic dysfunction, and age-dependent locomotor decline analogous to mammalian AD-related neurodegeneration [80,81,82]. The GeneSwitch/RU486 system permits temporally controlled activation of neuronal transgene expression in adult flies, minimizing developmental confounds [83,84,85]. In our study, RU486-treated flies exhibited progressive locomotor decline in the negative geotaxis climbing assay over 28 days, and WGE supplementation significantly improved climbing scores relative to the RU486-only group in a time- and dose-dependent manner, consistent with its BACE-1 inhibitory activity, antioxidant capacity, and putative anti-neuroinflammatory effects [71,80,81,82]. Brain BACE-1 activity measured fluorometrically in fly head lysates was significantly elevated by RU486 and progressively suppressed by *W. globosa* extract, providing direct biochemical evidence that in vitro BACE-1 inhibitory potency translates into the suppression of amyloidogenic enzyme activity in an intact organism [67,80,81,82,83]. Although *Drosophila* models cannot fully recapitulate the complexity of human AD, they offer rapid, cost-effective, and genetically validated in vivo evidence that supports further evaluation in mammalian systems.

Several limitations should be acknowledged. First, the in vitro findings were obtained from cell-free enzyme assays and should be interpreted as assay-specific evidence of inhibitory activity rather than proof of direct target engagement, selectivity, or therapeutic activity in vivo. In particular, the IC_50_ values of WGE against AChE, BChE, and BACE-1 represent concentrations of the total crude extract required to inhibit the respective enzyme activities under the experimental conditions; they do not indicate the concentration of individual active constituent(s) nor do they establish that comparable systemic or brain concentrations can be achieved after oral administration. The pharmacokinetics, oral bioavailability, metabolism, plasma and brain exposure, blood–brain barrier penetration, and dose–exposure relationships of WGE and its constituents were not determined. These factors are particularly important for chemically complex, phenolic- and lipid-containing botanical extracts, for which absorption, biotransformation, and central nervous system exposure may differ substantially from concentrations used in cell-free assays [45,57,58,59,62]. Second, the chemical analysis provided putative annotations of selected constituents based on HPLC-qTOF-MS data and relative peak areas, rather than definitive identification and absolute quantification of all compounds in WGE. Thus, the observed biological effects cannot be attributed exclusively to polyphenols, luteolin, apigenin, pinolenic acid, or any other individual constituent. The extract contains both phenolic and non-phenolic compounds, and its activity may reflect contributions from multiple constituents and potential additive, synergistic, or antagonistic interactions. Targeted quantification using authentic reference standards, confirmation of compound identities, testing of purified compounds and biologically relevant mixtures, and bioactivity-guided or synergy-directed fractionation are needed to identify the active constituent(s) and fraction(s) responsible for the observed effects. Complex botanical mixtures may exhibit multi-constituent interactions, and activity can be lost or altered following fractionation [86,87].

Fourth, the *Drosophila melanogaster* model provided preliminary organism-level evidence that dietary WGE and selected compounds were associated with changes in behavioral and biochemical endpoints. However, differences between flies and mammals in metabolism, neuroanatomy, immune biology, blood–brain barrier properties, protein interactions, and disease time course limit direct extrapolation to mammalian Alzheimer’s disease or human health outcomes. Furthermore, the study did not establish whether WGE constituents reached the fly brain, whether the measured changes in BACE-1 activity reflected direct enzyme inhibition or indirect physiological regulation, or whether these changes were causally related to behavioral outcomes. *Drosophila* models are useful for mechanistic and screening studies but do not fully reproduce the pathological and translational complexity of human AD [81,82,83]. Fifth, although WGE and the selected compounds did not produce overt adverse effects on fly survival or the behavioral endpoints assessed at the tested dietary concentrations, these results do not establish long-term safety or safety in mammals and humans. The present study did not evaluate mammalian-cell cytotoxicity, repeated-dose toxicity, organ-specific toxicity, reproductive or developmental toxicity, genotoxicity, drug-interaction potential, or safety pharmacology. The findings should therefore not be interpreted as evidence that WGE is safe for long-term human use. Finally, the phytochemical composition and biological activity of *W. globosa* may vary according to genotype, cultivation conditions, nutrient availability, environmental exposure, harvest stage, post-harvest processing, extraction method, and storage conditions. Standardized cultivation, traceable raw-material sourcing, validated extraction procedures, quantitative chemical fingerprinting, and batch-to-batch quality-control specifications will be required before reproducible nutraceutical development can be considered [7,9,75].

## 4. Materials and Methods

### 4.1. Plant Material and Chemicals

*Wolffia globosa* was obtained from an organic farm in Phra Nakhon Si Ayutthaya Province, Thailand. The sample was placed on a mesh, rinsed twice with tap water, and rinsed twice with deionized (DI) water. The sample was on the mesh during the whole washing process. Then, the sample was air-dried at room temperature for 2 h and further dried with a freeze dryer (PowerDry PL9000, Heto Lab Equipment, Allerød, Denmark). The freeze-dried sample was ground to a fine powder using a 600 W Philips grinder (Philips Electronics Co. Ltd., Jakarta, Indonesia). The obtained powders were stored at −40 °C in aluminum foil bags until further analyses. Hexane, dichloromethane, absolute ethanol, and methanol were purchased from Merck (Darmstadt, Germany). Reference standards, including gallic acid, quercetin, and (±)-6-hydroxy-2,5,7,8-tetramethylchromane-2-carboxylic acid (Trolox), were obtained from Sigma-Aldrich (St. Louis, MO, USA). Donepezil hydrochloride (≥98% purity) was purchased from Tokyo Chemical Industry (Tokyo, Japan). Enzyme substrates and assay reagents, including 5,5′-dithiobis-(2-nitrobenzoic acid) (DTNB), acetylthiocholine iodide (ATChI), butyrylthiocholine iodide (BTChI), α-amylase (from porcine pancreas), α-glucosidase (*Saccharomyces cerevisiae*), *p*-nitrophenyl-α-d-glucopyranoside (*p*NPG), BACE-1 fluorometric assay kit, and RU486 (mifepristone), were purchased from Sigma-Aldrich (St. Louis, MO, USA). Unless otherwise specified, all chemicals and reagents were of analytical grade.

### 4.2. Extraction Procedure

#### 4.2.1. Solvent Screening

Freeze-dried *W. globosa* powder (1.0 g) was separately extracted with four solvents of increasing polarity, hexane, dichloromethane, distilled water, and 70% (*v*/*v*) aqueous ethanol, using 30 mL of solvent at 50 °C for 120 min with continuous agitation. Extracts were vacuum filtered through Whatman No. 1 filter paper. Two sub-conditions were evaluated: (i) direct use of filtrate without evaporation, and (ii) rotary evaporation (40 °C) followed by re-dissolution of the dry residue in DMSO (final stock 10 mg/mL, ≤1% *v*/*v* DMSO in assay wells). Evaporated and re-dissolved extracts were used in all downstream bioactivity experiments. The solvent yielding the highest TPC and TFC was selected for subsequent optimization.

#### 4.2.2. Single-Factor Experiments

Single-factor experiments were conducted to delineate operating ranges for the Box–Behnken design (BBD) by varying one parameter at a time while holding all others constant at default settings (50% ethanol; solid-to-solvent ratio 1:30 g/mL; 50 °C; 60 min): (i) Ethanol concentration: 0, 20, 40, 60, 80% (*v*/*v*); (ii) Solid-to-solvent ratio: 1:30, 1:40, 1:50 (g/mL); (iii) Temperature: 30, 50, 70 °C; (iv) Extraction time: 15, 30, 45, 60 min. All conditions were performed in triplicate.

#### 4.2.3. Response Surface Methodology (RSM) Using Box–Behnken Design (BBD)

A four-factor, three-level BBD was applied with ethanol concentration (X_1_: 30, 50, 70% *v*/*v*), solid-to-solvent ratio (X_2_: 1:20, 1:30, 1:40 g/mL), extraction temperature (X_3_: 30, 45, 60 °C), and extraction time (X_4_: 20, 40, 60 min). All BBD and RSM analyses were conducted using Design-Expert version 13.0 (Stat-Ease, Inc., Minneapolis, MN, USA).

### 4.3. Determination of Total Phenolic Contents (TPCs), Total Flavonoid Contents (TFCs) and Total Tannin Contents (TTCs)

The TPCs of the extracts were assayed according to the Folin–Ciocalteu colorimetric assay described by [88], without modification. Following color development, absorbance was measured at 765 nm using a microplate reader. Gallic acid was used as the reference standard, and a calibration curve (10–200 mg/mL) was constructed to calculate the TPC. The results are expressed as milligrams of gallic acid equivalents per gram of dry weight (mg GAE/g DW).

The TFCs were measured using the aluminum chloride assay following the procedure reported by a previous study without modification [89]. The absorbance was recorded at 510 nm, and flavonoid concentrations were calculated from a quercetin standard curve (10–1000 mg/mL). The TFC is reported as milligrams of quercetin equivalents per gram of dry weight (mg QE/g DW).

The TTCs were evaluated using a modified Folin–Ciocalteu method based on the study [90]. Briefly, 25 µL of each extract was mixed with 50 µL of Folin–Ciocalteu reagent and allowed to react for 5 min before the addition of 200 µL sodium carbonate (Na_2_CO_3_). After incubation for 30 min at room temperature, the absorbance was measured at 700 nm. Tannic acid served as the calibration standard, and the tannin content was expressed as milligrams of tannic acid equivalents per gram of dry weight (mg TAE/g DW).

All absorbance measurements were performed using a BioTek microplate reader (BioTek Instruments, Inc., Winooski, VT, USA), and the data were processed with Gen5 software version 2.09.1.

### 4.4. Antioxidant Activity Assays

The antioxidant activity of WGE was evaluated by 2,2-diphenyl-1-picrylhydrazyl (DPPH) radical scavenging, oxygen radical absorbance capacity (ORAC), and ferric reducing antioxidant power (FRAP) assays, based on a previous protocol [91].

For the DPPH assay, the extract was mixed with freshly prepared ethanolic DPPH solution and incubated in the dark for 30 min at room temperature. The absorbance decrease was read at 520 nm in a microplate reader. The radical scavenging activity was calculated as relative to the control and expressed as Trolox equivalents (TE).

The ORAC assay was carried out using fluorescein as the fluorescent probe and 2,2′-azobis(2-amidinopropane) dihydrochloride as the peroxyl radical generator. Fluorescence was assayed using a fluorescence microplate reader at an excitation wavelength of 485 nm, and an emission wavelength of 528 nm. The ORAC activity was calculated from the net area under the fluorescence decay curve and expressed as µmol TE/g extract.

The FRAP assay was conducted using FRAP reagent containing acetate buffer, 2,4,6-tripyridyl-s-triazine, and ferric chloride. After incubation with the FRAP reagent at room temperature, the increase in absorbance resulting from the reduction of ferric (Fe^3+^) to ferrous (Fe^2+^) ions was measured at 595 nm. The reducing power was quantified using a Trolox calibration curve and expressed as µmol TE/g extract.

### 4.5. Enzyme Inhibitory Assays

The inhibitory activities against lipase, α-glucosidase, acetylcholinesterase (AChE), butyrylcholinesterase (BChE), and β-secretase (BACE-1) were tested according to a previous protocol with minor modifications [92]. Lipase inhibition was briefly assayed using *Candida rugosa* lipase (type VII, ≥700 U/mg) with 5,5′-dithiobis (2-nitrobenzoic acid)-*N*-phenacyl-4,5-dimethylthiazolium bromide as the substrate and 5,5′-dithiobis (2-nitrobenzoic acid) (DTNB) as the chromogenic reagent. α-Glucosidase inhibition was determined using α-glucosidase from *Saccharomyces cerevisiae* (type I, ≥10 U/mg protein) and *p*-nitrophenyl-α-D-glucopyranoside (pNPG) as a substrate. Acetylcholinesterase from *Electrophorus electricus* (1000 U/mg) was used for determining the AChE inhibitory activity, acetylthiocholine iodide (ATChI) was used as the substrate, and DTNB was used as the chromogenic reagent. Butyrylcholinesterase from equine serum (≥10 U/mg protein) was used for the study of the BChE inhibition activity, butyrylthiocholine iodide (BTChI) was used as the substrate and DTNB. The BACE-1 inhibitory activity was evaluated by using a commercial BACE-1 fluorescence resonance energy transfer (FRET) assay kit according to the manufacturer’s protocol. All enzyme inhibition assays were performed with the WGE as inhibitors. Absorbance or fluorescence was measured in a microplate reader. Lipase, AChE and BChE activities were measured at 412 nm, α-glucosidase activity at 405 nm, and BACE-1 activity at an excitation wavelength of 320 nm and emission wavelength of 405 nm. For IC_50_ determination, the WGE was tested at the following concentrations: AChE, 0.8, 1.25, 2.0, 2.5, 6.25, and 10 mg/mL; BChE, 0.8, 1.25, 2.0, 2.5, 6.25, and 10 mg/mL; BACE1, 0.5, 1.0, 1.25, 1.5, 2.0, and 2.5 mg/mL; and α-amylase, 1.25, 2.5, 3.5, 5.5, 6.5, and 12.5 mg/mL. All assays were carried out in 96-well plates, absorbance was measured using a BioTek microplate reader (BioTek Instruments, Inc., Winooski, VT, USA), and the data were analyzed with Gen5 software. Concentration–response curves (6–8 concentrations) were plotted and IC_50_ values determined where applicable.

### 4.6. Synergistic Interaction Assessment

Synergistic interactions between the optimized WGE and donepezil against AChE, BChE, and BACE-1 were evaluated followed a detailed procedure [91]. In brief, each agent was applied alone at approximately its IC_50_ concentration (WGE: 7300 µg/mL for AChE, 6500 µg/mL for BChE, 1600 µg/mL for BACE-1; donepezil: 1.5 µg/mL for AChE, 1.2 µg/mL for BChE, 0.6 µg/mL for BACE-1) and in combination. The experimental value (EV) was defined as the observed inhibition activity of each combination. The corresponding theoretical value (TV) for the expected additive effect was calculated as the sum of one-half of the inhibitory activity exerted by each individual component as follows:

$$\mathrm{TV}\;=\;\left(\frac{\mathrm{EV}\;\mathrm{of}\;\mathrm{synthetic}\;\mathrm{drug}}{2}\right)+\left(\frac{\mathrm{EV}\;\mathrm{of}\;\mathrm{an}\;\mathrm{extract}}{2}\right)$$

where drug and extract refer to the percentage of inhibition when each component was tested alone at the same concentration used in the combination.

The interaction of the extract and the pharmaceutical agent was classified by comparison of the EV to the TV. Synergistic interaction was assumed when the EV was >5% higher than the TV and antagonistic interaction was assumed when the TV was >5% higher than the EV. Differences of ±5% or less were additive.

### 4.7. Untargeted Metabolomic Profiling of Phytochemicals by High-Performance Liquid Chromatography–Quadrupole Time-of-Flight Tandem Mass Spectrometry (HPLC-QTOF-MS/MS)

The optimized WGE was reconstituted in absolute methanol and filtered through a 0.20 µm nylon membrane filter prior to analysis. An aliquot (2 µL) was injected onto an Acclaim™ RSLC 120 C18 analytical column (100 × 2.1 mm, 2.2 µm particle size, 120 Å pore size; Thermo Fisher Scientific, Waltham, MA, USA) coupled to a high-performance liquid chromatography (HPLC) system interfaced with a quadrupole time-of-flight tandem mass spectrometer (TripleTOF^®^ 6600+, SCIEX, Framingham, MA, USA). Chromatographic separation was performed using a binary mobile phase consisting of 0.1% formic acid in water (mobile phase A) and 0.1% formic acid in acetonitrile (mobile phase B) under gradient elution. The gradient program was as follows: 0.00–1.00 min, 1% B; 1.00–21.00 min, 1–80% B; 21.00–21.10 min, 80–95% B; 21.10–25.00 min, 95% B; 25.00–25.10 min, 95–1% B; and 25.10–30.00 min, 1% B. The flow rate was maintained at 0.40 mL/min, resulting in a total run time of 30 min, according to a previously established method [93]. Mass spectrometric data were acquired in both positive and negative electrospray ionization (ESI) modes over an *m*/*z* range of 100–1000. Instrument parameters were set as follows: ion spray voltage, 5.5 kV; source temperature, 500 °C; nebulizer gas (GS1), 50 psi; heater gas (GS2), 60 psi; declustering potential, 80 V; and collision energy, 30 ± 15 eV. Putative phytochemical identification from WGE was achieved by matching the acquired MS/MS spectra with entries in the National Institute of Standards and Technology (NIST) Mass Spectral Library and the SCIEX High-Resolution Natural Products MS/MS Spectral Library using SCIEX OS software version 3.1.0 (SCIEX, Framingham, MA, USA). A representative base peak chromatogram is presented in Supplementary Figure S8.

### 4.8. Targeted Phytochemical Analysis Using Liquid Chromatography-Electrospray Ionization-Tandem Mass Spectrometry (LC-ESI-MS/MS)

Sample preparation for LC-ESI-MS/MS analysis was performed under two conditions: (i) acid hydrolysis (6 N HCl, reflux 100 °C, 2 h, followed by liquid–liquid extraction with ethyl acetate) and (ii) non-acid hydrolysis (direct injection). Briefly, the extract was prepared at a concentration of 10 mg/mL in 62.5% (*v*/*v*) methanol containing tert-butylhydroquinone (TBHQ). The solution was incubated at 80 °C for 2 h and subsequently filtered through a 0.22 µm polyethersulfone (PES) membrane before chromatographic analysis. Phenolic compounds were separated using an Accucore RP-MS column (2.1 × 100 mm, 2.6 µm; Thermo Fisher Scientific, Bremen, Germany) coupled to a Dionex Ultimate 3000 ultra-high-performance liquid chromatography (UHPLC) system equipped with a diode-array detector and a TSQ Quantis triple quadrupole mass spectrometer (Thermo Fisher Scientific; Bremen; Germany). Data acquisition and instrument control were carried out using Chromeleon chromatography software (version 7.2.9.11323; Thermo Fisher Scientific, Bremen, Germany). The mobile phase consisted of acetonitrile (solvent A) and 0.1% (*v*/*v*) formic acid in Milli-Q water (18.2 MΩ·cm at 25 °C; solvent B). Chromatographic separation was performed at a constant flow rate of 0.5 mL/min using the following gradient program: 10–80% solvent A over 0.0–8.0 min, 80–10% solvent A over 8.0–8.1 min, followed by re-equilibration at 10% solvent A from 8.1 to 10.0 min. Identification and quantification of phenolic constituents were performed using authentic reference standards following the method reported by Sirichai et al. [88]. A total of 24 phenolic compounds, comprising 12 phenolic acids and 12 flavonoids, were included in the analysis. The phenolic acid standards consisted of gallic acid, 3,4-dihydroxybenzoic acid, 4-hydroxybenzoic acid, chlorogenic acid, caffeic acid, vanillic acid, syringic acid, p-coumaric acid, ferulic acid, sinapic acid, cinnamic acid, and rosmarinic acid. The flavonoid standards included rutin, (−)-epigallocatechin gallate, hesperidin, myricetin, luteolin, quercetin, naringenin, kaempferol, apigenin, genistein, isorhamnetin, and galangin. These analytical standards were obtained from Tokyo Chemical Industry (Tokyo, Japan), Sigma-Aldrich (St. Louis, MO, USA), Extrasynthese (Genay, France), and Wuhan ChemFaces Biochemical Co., Ltd. (Wuhan, China).

### 4.9. Molecular Docking and Molecular Dynamic (MD) Simulation

Luteolin, apigenin, naringenin, caffeic acid, isovitexin, and schaftoside were docked against crystal structures of human acetylcholinesterase (PDB: 7E3H), human butyrylcholinesterase (PDB: 4TPK), and human BACE-1 (PDB: 6EQM) retrieved from the RCSB Protein Data Bank. Protein preparation was performed in AutoDockTools 1.5.7. Docking was performed using AutoDock Vina 1.2.x [68] with exhaustiveness = 32. Grid boxes were centered on the respective active site coordinates: AChE (7E3H): center X = −50.968, Y = 37.287, Z = −32.71; grid 48 × 46 × 54 angstroms (active site) and 100 × 96 × 104 angstroms (whole macromolecule). BChE (4TPK): center X = −5.000, Y = 10.000, Z = 15.000; grid 30 × 30 × 30 angstroms (active site). BACE-1 (6EQM): center X = 22.559, Y = 74.559, Z = 17.394; grid 36 × 26 × 36 angstroms (active site). Binding interactions were visualized using PyMOL v.2.x and LigPlot+ v.2.2.

MD simulation was then performed using GROMACS 2018 to evaluate the binding and binding stability of the enzyme–phytochemical complexes. The complexes were parameterized using the CHARMM36 force field, solvated in a cubic box with TIP3P water molecules, and neutralized by adding counter ions. The root mean square deviation (RMSD) and root mean square fluctuation (RMSF) were analyzed over a 1000 ps simulation to assess the structural stability and dynamic behavior of the enzyme–phytochemical complexes.

### 4.10. Drosophila melanogaster In Vivo Experiments

#### 4.10.1. Fly Strains and Husbandry

Flies carrying human APPs and the BACE-1 construct (BDSC 56756) were obtained from the Bloomington Stock Center at Indiana University and cultured on Formula 4–24 blue^®^ medium (Carolina, Burlington, NC, USA). For AD stimulation, one hundred one-day-old adult flies were fed with food supplemented with RU486 (mifepristone) at 20 µg/mL to induce APP and BACE-1 transgene expression via the GeneSwitch system [83]. Treatment groups were: (1) Control (no AD control)—standard diet; (2) RU486 (AD model)—standard diet + RU486; (3) WGE treatment groups—RU486 diet + WGE at 62.5 to 500 µg/mL; (4) luteolin treatment groups—RU486 diet + luteolin at 0.05 and 1.00 µg/mL; (5) apigenin treatment groups—RU486 diet + apigenin at 0.20 and 0.40 µg/mL; (6) donepezil positive control—RU486 diet + donepezil at 10 µM. Flies were cultured at 28 °C, and a 12-h/12-h light–dark cycle in constant climate chambers with an ICH-compliant light source (BINDER GmbH, Tuttlingen, Germany) for 28 days. To keep the food fresh, it was changed every three days.

#### 4.10.2. Climbing Assay

Locomotor performance was assessed using the climbing assay at days 7, 14, 21, and 28 of treatment. Flies in each experiment were divided into four groups and placed in a glass tube without anesthesia. Following mechanical stimulation (tapping the vial three times), the percentage of flies reaching or exceeding a line marked were subsequently calculated as described [94]. All assessments were performed between 09:00 and 12:00 h to minimize circadian variability.

#### 4.10.3. Brain BACE-1 Activity Assay

At each time point, flies (*n* = 30) were anaesthetized, and heads were collected rapidly using the centrifugation technique. Brain homogenates were prepared by T-PER™ Tissue Protein Extraction Reagent (Thermo Fisher Scientific, Waltham, MA, USA), followed by centrifugation at 10,000× *g* for 5 min at 4 °C. Protein concentration was determined by the BCA protein assay (Thermo Fisher Scientific, Waltham, MA, USA), and BACE-1 activity was quantified using the BACE-1 assay kit described in Section 4.5. BACE-1 activity in fly brains was expressed as pmol/g protein.

### 4.11. Statistical Analysis

All experiments were performed in triplicate (*n* = 3) unless otherwise stated, and results are expressed as mean ± standard deviation (SD). Statistical significance was assessed by one-way ANOVA followed by Duncan’s multiple range test (*p* < 0.05). For fly group comparisons, the unpaired Student’s *t*-test was applied with significance thresholds of *p* < 0.05 (*), *p* < 0.01 (**), *p* < 0.001 (***), and *p* < 0.0001 (****). BBD and RSM analysis were performed using Design-Expert v.13.0 (Stat-Ease Inc., Minneapolis, MN, USA). IC_50_ values were determined by non-linear regression.

## 5. Conclusions

This study provides a comprehensive, multi-phase bioactive characterization of *Wolffia globosa* by integrating systematic extraction optimization, multi-target enzyme inhibition profiling, single-dose combination testing with donepezil, high-resolution multi-platform metabolomics, computational molecular docking, and *Drosophila melanogaster* in vivo neuroprotective assessment. Using Box–Behnken design-guided response surface methodology, an optimized extraction protocol (32% ethanol, 60 °C, 28 min, 1:40 g/mL) was established, yielding polyphenol-containing extracts with validated TPCs of 15.17 ± 0.27 mg GAE/g DW and TFCs of 22.34 ± 1.71 mg QE/g DW.

The optimized extract exhibited a high antioxidant capacity (ORAC: 4374 µmol TE/g DW), potent and selective BACE-1 inhibition (IC_50_: 1.59 mg/mL), and greater-than-additive enhancement of donepezil’s inhibitory activity against AChE, BChE, and BACE-1 at the tested combination dose, with BACE-1 inhibition approaching ~82%—approximately 30 percentage points above the theoretical additive value. Multi-platform metabolite profiling confirmed a rich bioactive matrix encompassing luteolin, apigenin, caffeic acid, isovitexin, schaftoside, pinolenic acid, tocopherols, and phytosterols, while molecular docking supported strong binding of luteolin and apigenin (free energies ~ −8.9 to −10.2 kcal/mol) to all three AD-related enzyme targets.

Importantly, *Drosophila* in vivo experiments demonstrated that *W. globosa* extract supplementation significantly attenuated RU486-induced locomotor decline and reduced brain BACE-1 activity in a time- and dose-dependent manner over 28 days, providing initial organismal-level evidence of neuroprotective potential for this species. Beyond its established protein and micronutrient credentials, *W. globosa* thus emerges as a promising polyfunctional food ingredient positioned at the interface of sustainable food production and nutraceutical development for cognitive health. With further validation in mammalian models, pharmacokinetic characterization, and formulation optimization, *W. globosa* may offer genuine potential as a dietary–pharmacological adjunct for Alzheimer’s disease and related neurodegenerative conditions, as well as a high-value functional food ingredient for aging populations worldwide.

## Figures and Tables

**Table 1:** Effects of different ethanol concentrations on the total phenolic contents (TPCs) and total flavonoid contents (TFCs) of *W. globosa* extracts (controlled variables: 50 °C, 60 min, ratio 1:30 g/mL).

Independent Variable (EtOH Concentration, %)	Dependent Variables	
	TPCs (mg GAE/g DW)	TFCs (mg QE/g DW)
0	11.49 ± 0.13 ^b^	9.45 ± 0.69 ^b^
20	12.40 ± 0.06 ^a^	9.95 ± 0.56 ^b^
40	11.67 ± 0.10 ^b^	11.35 ± 0.37 ^a^
60	12.72 ± 0.32 ^a^	11.87 ± 0.76 ^a^
80	11.05 ± 0.23 ^c^	13.59 ± 3.00 ^a^

Superscript letters (a–c) indicate statistically significant differences within each column (*p* < 0.05).

**Table 2:** Effects of different solid-to-solvent ratios on the total phenolic contents (TPCs) and total flavonoid contents (TFCs) of *W. globosa* extracts (controlled variables: 50 °C, 60 min, 50% ethanol).

Independent Variable (Extraction Ratio, g:mL)	Dependent Variables	
	TPCs (mg GAE/g DW)	TFCs (mg QE/g DW)
1:30	12.64 ± 0.56 ^b^	10.40 ± 1.04 ^a^
1:40	11.98 ± 0.14 ^c^	10.55 ± 0.80 ^a^
1:50	13.04 ± 0.11 ^a^	8.23 ± 0.50 ^b^

Superscript letters (a–c) indicate statistically significant differences within each column (*p* < 0.05).

**Table 3:** Effects of different temperatures on the total phenolic contents (TPCs) and total flavonoid contents (TFCs) of *W. globosa* extracts (controlled variables: 50 °C, 50% ethanol, ratio 1:30 g/mL).

Independent Variable (Temperature, °C)	Dependent Variables	
	TPCs (mg GAE/g DW)	TFCs (mg QE/g DW)
30	12.37 ± 0.03 ^ab^	14.59 ± 0.45 ^b^
50	12.13 ± 0.14 ^b^	14.97 ± 0.24 ^b^
70	12.51 ± 0.14 ^a^	19.75 ± 0.77 ^a^

Superscript letters (a,b) indicate statistically significant differences within each column (*p* < 0.05).

**Table 4:** Effects of different extraction times on the total phenolic contents (TPCs) and total flavonoid contents (TFCs) of *W. globosa* extracts (controlled variables: 50 °C, 50% ethanol, ratio 1:30 g/mL).

Independent Variable (Time, min)	Dependent Variables	
	TPCs (mg GAE/g DW)	TFCs (mg QE/g DW)
15	11.81 ± 0.25 ^bc^	12.41 ± 0.41 ^bc^
30	11.64 ± 0.06 ^c^	11.70 ± 0.97 ^c^
45	12.27 ± 0.06 ^a^	13.37 ± 0.11 ^ab^
60	12.04 ± 0.18 ^ab^	14.08 ± 0.40 ^a^

Superscript letters (a–c) indicate statistically significant differences within each column (*p* < 0.05).

**Table 5:** Coded independent variables (ethanol concentration, solid-to-solvent ratio, extraction temperature, and extraction time) and dependent variables (total flavonoid contents (TFCs) and total phenolic contents (TPCs)) derived from the Box–Behnken design (BBD).

Run	X_1_ Ethanol (%*v*/*v*)		X_2_ Ratio (g: mL)		X_3_ Temperature (°C)		X_4_ Time (min)		TPCs (mg GAE/g DW)	TFCs (mg QE/g DW)
	Coded	Uncoded	Coded	Uncoded	Coded	Uncoded	Coded	Uncoded	Experimental	Experimental
1	1	70	−1	20	0	45	0	40	8.09 ± 0.48	9.95 ± 0.28
2	0	50	−1	20	1	60	0	40	8.16 ± 0.61	14.40 ± 0.80
3	1	70	1	40	0	45	0	40	10.00 ± 0.38	9.73 ± 1.05
4	−1	30	0	30	0	45	1	60	11.51 ± 0.34	17.88 ± 0.32
5	0	50	−1	20	0	45	−1	20	9.02 ± 0.02	14.40 ± 1.06
6	0	50	0	30	−1	30	1	60	9.58 ± 0.70	18.71 ± 1.38
7	0	50	−1	20	−1	30	0	40	9.15 ± 0.51	18.34 ± 1.38
8	0	50	0	30	1	60	−1	20	9.84 ± 0.90	16.84 ± 0.50
9	0	50	1	40	0	45	1	60	11.80 ± 0.52	19.70 ± 1.41
10	−1	30	0	30	0	45	−1	20	12.70 ± 0.03	17.81 ± 0.74
11	1	70	0	30	1	60	0	40	10.59 ± 0.34	11.53 ± 0.78
12	1	70	0	30	0	45	1	60	8.60 ± 0.87	9.90 ± 0.79
13	1	70	0	30	−1	30	0	40	7.94 ± 0.48	10.59 ± 0.82
14	0	50	−1	20	0	45	1	60	9.58 ± 0.72	17.21 ± 0.91
15	−1	30	1	40	0	45	0	40	11.77 ± 0.19	15.48 ± 0.84
16	−1	30	0	30	1	60	0	40	14.30 ± 0.19	23.55 ± 0.70
17	1	70	0	30	0	45	−1	20	7.75 ± 0.27	9.32 ± 0.67
18	0	50	0	30	0	45	0	40	9.33 ± 0.21	19.45 ± 0.45
19	0	50	0	30	0	45	0	40	9.35 ± 0.36	18.28 ± 1.50
20	−1	30	−1	20	0	45	0	40	11.95 ± 0.14	15.56 ± 0.71
21	0	50	1	40	−1	30	0	40	10.96 ± 0.44	16.92 ± 0.85
22	−1	30	0	30	−1	30	0	40	12.57 ± 0.33	20.49 ± 0.64
23	0	50	0	30	−1	30	−1	20	9.64 ± 0.36	18.09 ± 1.44
24	0	50	1	40	1	60	0	40	12.69 ± 1.18	17.11 ± 1.29
25	0	50	0	30	1	60	1	60	11.82 ± 0.86	16.14 ± 1.05
26	0	50	0	30	0	45	0	40	10.25 ± 0.60	18.24 ± 0.66
27	0	50	1	40	0	45	−1	20	11.44 ± 0.19	19.67 ± 1.21

Experimental data are shown as mean ± standard deviation (SD) of triplicate experiments (*n* = 3). Superscript letters in each column indicate significantly different TPCs or TFCs in each run condition from BBD at *p <* 0.05, calculated by one-way analysis of variance (ANOVA) and Duncan’s multiple comparison test. GAE = gallic acid equivalent, QE = quercetin equivalent, DW = dry weight.

**Table 6:** Analysis of variance, regression coefficients, and *p*-value of the second-order polynomial models for the total phenolic contents (TPCs) and total flavonoid contents (TFCs).

Source	TPCs					TFCs				
	Sum of Squares	df	Mean Square	*p*-Value	Significant	Sum of Squares	df	Mean Square	*p*-Value	Significant
Model	67.33	14	4.81	0.0013	**	310.11	14	22.15	0.0054	**
X_1_	39.74	1	39.74	<0.0001	****	206.37	1	206.37	<0.0001	****
X_2_	13.45	1	13.45	0.0011	**	6.35	1	6.35	0.2692	
X_3_	4.78	1	4.78	0.0261	*	1.06	1	1.06	0.6451	
X_4_	0.5225	1	0.5225	0.4181		0.9611	1	0.9611	0.6601	
X_1_X_2_	1.10	1	1.10	0.2466		0.0052	1	0.0052	0.9742	
X_1_X_3_	0.2130	1	0.2130	0.6021		1.12	1	1.12	0.6351	
X_1_X_4_	1.05	1	1.05	0.2579		0.0662	1	0.0662	0.9078	
X_2_X_3_	1.85	1	1.85	0.1406		4.27	1	4.27	0.3605	
X_2_X_4_	0.0093	1	0.0093	0.9126		1.93	1	1.93	0.5351	
X_3_X_4_	1.03	1	1.03	0.2609		0.4369	1	0.4369	0.7664	
X_1_^2^	2.43	1	2.43	0.0959		71.43	1	71.43	0.0022	**
X_2_^2^	0.4138	1	0.4138	0.4698		13.21	1	13.21	0.1204	
X_3_^2^	1.97	1	1.97	0.1292		0.2213	1	0.2213	0.8323	
X_4_^2^	0.0675	1	0.0675	0.7682		2.43	1	2.43	0.4875	
Residual	8.91	12	0.7429			56.74	12	4.73		
Lack of Fit	8.36	10	0.8362	0.2739	ns	55.80	10	5.58	0.0804	ns
Pure Error	0.5527	2	0.2763			0.9432	2	0.4716		
Cor Total	76.25	26				366.85	26			
R^2^	0.8831					0.8453				
Adjusted R^2^	0.7467					0.6649				

X_1_; A = % Ethanol, X_2_; B = Ratio, X_3_; C = Temperature, X_4_; D = Time. Statistical analyses were determined by one-way analysis of variance (ANOVA). *, *p* < 0.05; **, *p* < 0.01; ****, *p* < 0.0001; NS: not statistically significant.

**Table 7:** TPCs, TFCs, TTCs, and antioxidant activity of optimized WGE (32% EtOH, ratio 1:40, 60 °C, 28 min).

TPCs (mg GAE/g DW)	TFCs (mg QE/g DW)	TTCs (mg TAE/g DW)	Antioxidant Activity		
			ORAC (µmol TE/g DW)	FRAP (µmol TE/g DW)	DPPH (µmol TE/g DW)
15.17 ± 0.27	22.34 ± 1.71	21.13 ± 0.22	4374.33 ± 395.65	210.64 ± 1.96	136.95 ± 4.22

**Table 8:** Inhibitory activity of the optimized *W. globosa* extract (WGE) against enzymes involved in AD.

Enzymes	% Inhibition (2 mg/mL)	Half Maximal Inhibitory Concentration IC_50_ (mg/mL)
Acetylcholine esterase (AChE)	13.88 ± 1.38	7.42 ± 0.16
Butylcholine esterase (BChE)	15.61 ± 1.28	6.55 ± 0.24
Beta-secretase (BACE1)	56.94 ± 3.05	1.59 ± 0.11

**Table 9:** Combined inhibitory effects of WGE and donepezil on AChE, BChE, and BACE-1.

Enzymes	Compounds	Experimental	Theoretical	Result
AChE	Donepezil (1.5µg/mL)	48.10 ± 1.23	-	Supra-additive at tested dose
	WGE (7300 µg/mL)	49.35 ± 1.74	-	
	WGE + Donepezil	60.32 ± 0.56	48.72	
BChE	Donepezil (1.2 µg/mL)	50.10 ± 1.09	-	Supra-additive at tested dose
	WGE (6500 µg/mL)	49.35 ± 1.74	-	
	WGE + Donepezil	77.02 ± 0.19	49.72	
BACE-1	Donepezil (0.6 µg/mL)	52.11 ± 1.17	-	Supra-additive at tested dose
	WGE (1600 µg/mL)	50.72 ± 0.67	-	
	WGE + Donepezil	82.16 ± 1.88	51.42	

**Table 10:** HPLC-qTOF metabolomics of optimized *W. globosa* (negative mode-cut off library score 95).

No.	RT (min)	Compound	Mode	Molecular Mass	Precursor Mass	Library Score	Area	%Area
1	20.89	Pinolenic acid	M−	277.2197	277.2200	90.8	29,590,000	64.82
2	14.42	1-Palmitoyl-2-hydroxy-sn-glycero-3-phospho-(1′-rac-glycerol)	M−	483.2773	483.2770	94.5	12,050,000	26.40
3	3.92	Caffeic acid	M−H−	179.0361	179.0360	94.0	1,621,000	3.55
4	10.36	Luteolin	M−H−	285.0413	285.0410	97.8	1,231,000	2.70
5	27.98	Ellagic acid	M−	300.9982	300.9980	92.1	589,500	1.29
6	15.10	1-Oleoyl-l-α-lysophosphatidic acid	M−	435.2517	435.2510	96.5	252,000	0.55
7	5.22	p-Coumaric acid	M−	163.0408	163.0410	96.0	168,700	0.37
8	6.54	Isovitexin	M+FA-H−	430.1926	431.1930	98.8	44,240	0.10
9	5.55	Isovitexin	M−	431.0760	431.0760	98.8	41,260	0.09
10	19.65	Cholesterol 3-sulfate	M−	465.3187	465.3190	96.9	27,990	0.06
11	17.66	Gingerglycolipid B	M−	723.2899	723.2900	96.3	27,620	0.06
12	7.22	Isoschaftoside	M−	563.2859	563.2860	98.5	5339	0.01

Remarks: All annotations are putative identifications based on accurate mass together with MS/MS spectral-library matching (SCIEX/NIST), without confirmation by authentic reference standards. Mode denotes the observed adduct/ionization form; %Area is expressed relative to the total annotated peak area.

**Table 11:** HPLC-qTOF metabolomics of optimized *W. globosa* (positive mode-cut off library score 95).

No.	RT (min)	Compound	Mode	Molecular Mass	Precursor Mass	Library Score	Area	%Area
1	6.99	Isovitexin	M+K+	433.1185	433.1180	96.5	15,820,000	26.35
2	5.97	Schaftoside	M+	565.1672	565.1670	96.4	7,763,000	12.93
3	18.27	1-Palmitoyl-sn-glycero-3-phosphocholine	M+	496.3534	496.3534	99.0	6,550,000	10.91
4	16.05	Monolinolenin	M+	353.2784	353.2784	98.4	3,658,000	6.09
5	4.02	3-Indoleacrylic acid	M+	188.0796	188.0800	93.7	3,059,000	5.10
6	17.21	13-Keto-9*Z*,11*E*-octadecadienoic acid	M+H+	295.2344	295.2344	94.2	2,723,000	4.54
7	3.94	5′-*S*-Methyl-5′-thioadenosine	M+	298.1097	298.1100	96.9	2,362,000	3.93
8	2.30	Phenylalanine	M+	166.0950	166.0950	99.8	2,082,000	3.47
9	19.52	1,2-Dioleoyl-sn-glycero-3-phosphatidylcholine	M+	786.6023	786.6023	99.6	1,571,000	2.62
10	1.41	Adenosine	M+	268.1154	268.1150	100.0	1,544,000	2.57
11	1.49	Isoleucine	M+	132.1099	132.1100	99.0	1,401,000	2.33
12	23.77	Phytanic acid	M+	313.2793	313.2793	92.9	1,329,000	2.21
13	26.16	Friedelin	M+	427.3938	427.3938	98.4	1,311,000	2.18
14	1.06	l-Valine	M+	118.0923	118.0920	98.8	1,241,000	2.07
15	24.76	(+)-α-Tocopherol	M+	431.3566	431.3566	94.0	1,113,000	1.85
16	1.41	Adenine	M+	136.0676	136.0680	94.5	1,036,000	1.73
17	3.97	l-Tryptophan	M+H+	205.1032	205.1030	94.8	986,500	1.64
18	26.68	1-Palmitoyl-2-linoleoyl-sn-glycero-3-phosphocholine	M+	758.5730	758.5730	93.7	586,900	0.98
19	23.34	1-Stearoyl-rac-glycerol	M+	359.3209	359.3209	94.9	532,200	0.89
20	1.02	l-Pipecolic acid	M+	130.0979	130.0980	96.0	447,800	0.75
21	1.41	l-Tyrosine	M+NH_4_+	182.0870	182.0870	93.1	364,400	0.61
22	17.24	1-Monolinoleoyl-rac-glycerol	M+CH_3_OH+H+	355.2900	355.2900	91.0	356,100	0.59
23	9.14	Luteolin	M+	287.0682	287.0680	100.0	346,500	0.58
24	6.79	Hyperin	M+	465.1104	465.1100	100.0	234,900	0.39
25	8.72	Daidzein	M+	255.0769	255.0770	95.4	233,100	0.39
26	18.87	1-Oleoyl-sn-glycero-3-phosphocholine	M+	552.3635	552.3635	98.9	211,600	0.35
27	1.32	Nicotinic acid	M+	124.0511	124.0510	98.0	184,100	0.31
28	24.94	Campesterol	M+H+	383.3727	383.3727	95.2	170,500	0.28
29	4.25	1*H*-Indole-4-carboxaldehyde	M+	146.0659	146.0660	95.2	135,400	0.23
30	0.98	Proline	M+	116.0747	116.0750	98.5	132,500	0.22
31	21.17	1-Palmitoyl-2-azelaoylphosphatidylcholine	M+	666.4364	666.4364	98.3	129,200	0.22
32	2.53	Pantothenic acid	M+H+	220.1267	220.1270	94.7	113,600	0.19
33	1.24	Glutamic acid	M+	148.1127	148.1130	98.9	79,650	0.13
34	0.85	Histidine	M+	156.0831	156.0830	99.0	77,440	0.13
35	12.45	Farnesal	M+	221.1594	221.1594	92.4	57,500	0.10
36	0.85	l-Lysine	M+	147.1177	147.1180	92.3	51,290	0.09
37	14.15	Monolinolenin	M+CH_3_OH+H+	353.2742	353.2742	94.4	28,590	0.05
38	8.88	Glycitein	M+	285.0864	285.0860	93.4	9173	0.02

Remarks: All annotations are putative identifications based on accurate mass together with MS/MS spectral-library matching (SCIEX/NIST), without confirmation by authentic reference standards. Mode denotes the observed adduct/ionization form; %Area is expressed relative to the total annotated peak area.

**Table 12:** LC-MS/MS-based putative identification of selected compounds in the optimized *W. globosa* extract.

Type of Hydrolysis	Compounds (µg/mL)	
Non-acid hydrolysis	Luteolin	12.18 ± 0.17
	Caffeic acid	1.69 ± 0.01
Acid hydrolysis	Luteolin	1.98 ± 0.01
	Apigenin	0.77 ± 0.05
	Naringenin	0.32 ± 0.01

**Table 13:** Estimated free binding energy, numbers of hydrogen bonds, and interaction area of luteolin, caffeic acid, apigenin, naringenin, isovitexin and schaftoside against human AChE (PDB: 7E3H) active site or whole enzyme.

Chemical Compounds	Interactions Centered on Active Site			Interactions Centered on Macromolecule		
	Estimated Free Binding Energy (kcal/mol)	Number of Hydrogen Bonds	Interactions with Enzyme	Estimated Free Binding Energy (kcal/mol)	Number of Hydrogen Bonds	Interactions with Enzyme
Luteolin	−10.243	3	SER203, ALA204, ARG296, TYR337, TYR341	−10.213	5	GLY121, SER203, ALA204, SER293, PHE295, ARG296, TYR337, TYR341
Caffeic acid	−7.503	1	TRP86, GLY120, GLY122, ALA204, GLY448	−7.481	3	TRP86, GLY120, GLY122, GLU202, ALA204, GLY205, HIS447, GLY448
Apigenin	−9.883	2	GLY121, GLY122, SER203, ALA204, PHE295, TYR337	−9.885	2	GLY121, GLY122, PHE123, SER203, ALA204, SER293, PHE295, TYR337
Naringenin	−10.059	1	ALA204, SER293, PHE295	−9.972	2	GLY121, ALA204, SER293, PHE295, ARG296
Isovitexin	−10.137	2	TRP86, VAL294, PHE295, TYR337	−9.820	3	THR75, GLU84, TRP86, GLU202, GLN291, VAL294, PHE295, TYR337, TRP439
Schaftoside	−7.169	1	PHE297, TYR337, PHE338,	−8.630	3	TYR72, ASP74, ARG296, PHE297, TYR337, PHE338, LEU339, VAL340, TYR341

Receptor: AChE (PDB: 7E3H). The grid box was set to 48 × 46 × 54 and 100 × 96 ×104 for interactions centered on the active site and macromolecule, respectively. For center coordinates, the grid box was arranged at X = −50.968, Y = 37.287, and Z = −32.71 and X = −54.36, Y = 32.817, and Z = −28.775 for interactions centered on the active site and macromolecule, respectively, with a spacing of 0.400 Å.

**Table 14:** Estimated free binding energy, numbers of hydrogen bonds, and interaction area of luteolin, caffeic acid, apigenin, naringenin, isovitexin and schaftoside against human BChE (PDB: 4TPK) active site or whole enzyme.

Chemical compounds	Interactions Centered on Active Site			Interactions Centered on Macromolecule		
	Estimated Free Binding Energy (kcal/mol)	Number of Hydrogen Bonds	Interactions with Enzyme	Estimated Free Binding Energy (kcal/mol)	Number of Hydrogen Bonds	Interactions with Enzyme
Luteolin	−9.462	2	ASN68, GLY115, GLY116, GLY117, GLY121, GLU197	−9.410	3	ASN68, ASP70, GLY115, GLY116, GLY117, GLY121, GLU197, GLY439
Caffeic acid	−6.532	1	GLY116, GLU 197, SER 198, HIS 438	−6.515	2	GLY115, GLY116, THR122, GLU 197, SER 198, HIS438
Apigenin	−9.164	2	GLY78, GLY115, GLY116, THR122, VAL331HIS438, GLY439	−9.169	2	GLY78, GLY115, GLY116, THR122, GLU197, VAL331, ALA328
Naringenin	−9.062	2	ASN68, HIS438, GLY439, GLY116	−9.084	2	ASN68, GLY115, GLY116, GLN 119
Isovitexin	−9.319	1	GLY115, GLY116, GLY117, GLN119, THR120	−7.540	2	GLY115, GLY116, GLY117, PHE118, GLN119, THR120, GLY121, TYR128, GLU197
Schaftoside	−6.952	1	GLY115, GLY116, TRP430	−6.233	2	ASN83, PRO84, GLY115, GLY116, TRP430, MET437, TYR440, ILE442

Receptor: BChE (PDB: 4TPK). Grid box was set to 30 × 30 × 30 and 68 × 80 × 80 for interactions centered on the active site and macromolecule, respectively. For center coordinates, grid box was arranged at X = −5.000, Y = 10.000, and Z = 15.000 and X = −5.000, Y = 10.000, and Z = 15.000 for interactions centered on the active site and macromolecule, respectively, with the spacing of 0.400 Å.

**Table 15:** Estimated free binding energy, numbers of hydrogen bonds, and interaction area of luteolin, caffeic acid, apigenin, naringenin, isovitexin and schaftoside against human BACE-1 (PDB: 6EQM) active site or whole enzyme.

Chemical Compounds	Interactions Centered on Active Site			Interactions Centered on Macromolecule		
	Estimated Free Binding Energy (kcal/mol)	Number of Hydrogen Bonds	Interactions with Enzyme	Estimated Free Binding Energy (kcal/mol)	Number of Hydrogen Bonds	Interactions with Enzyme
Luteolin	−8.867	2	THR72, GLN73, THR329	−8.952	4	ASP32, GLY34, ASN37, THR72, GLN73, PHE108, THR329
Caffeic acid	−7.703	3	GLY34, TYR71, GLN73, ASP106, LYS107	−7.561	5	ASP32, GLY34, SER36, ASN37, TYR71, GLN73, LYS75, ASP106, LYS107
Apigenin	−8.664	2	THR72, GLN73, ASP106, LYS107, GLY230	−8.808	4	GLY34, ASN37, THR72, GLN73, ASP106, LYS107, GLY230, SER328
Naringenin	−8.760	3	GLY 34, ASN 37,THR72, GLN73, PHE108, THR329, GLY330	−8.763	3	GLY 34, ASN 37, THR72, GLN73, LYS107, PHE108, THR329, GLY330
Isovitexin	−7.546	1	SER35, PHE47, THR72, GLN73, GLY74	−6.660	1	SER35, PHE47, TYR71, THR72, GLN73, GLY74, LYS75
Schaftoside	−7.210	2	GLY11, GLN12, THR72, GLN73, GLY74, LYS75, TRP76	−6.100	2	GLY11, GLN12, PRO70, TYR71, THR72, GLN73, GLY74, LYS75, TRP76, LYS107

Receptor: BACE-1 (PDB: 6EQM). The grid box was set to 36 × 26 × 36 and 98 × 96 × 98 for interactions centered on the active site and macromolecule, respectively. For center coordinates, the grid box was arranged at X = 22.559, Y = 74.559, and Z = 17.394 and X = 21.668, Y = 72.397, and Z = 15.415 for interactions centered on the active site and macromolecule, respectively, with a spacing of 0.400 Å.

## Data Availability

Data are contained within this article and the Supplementary Materials.

## Supplementary Materials

The following supporting information can be downloaded at: https://www.mdpi.com/article/10.3390/ijms27188266/s1.

## References

[1] Springmann M., Clark M., Mason-D’Croz D., Wiebe K., Bodirsky B.L., Lassaletta L., Vries W.d., Vermeulen S.J., Herrero M., Carlson K.M. (2018). Options for keeping the food system within environmental limits. Nature.

[2] Willett W., Rockström J., Loken B., Springmann M., Lang T., Vermeulen S., Garnett T., Tilman D., DeClerck F., Wood A. (2019). Food in the Anthropocene: The EAT-Lancet Commission on healthy diets from sustainable food systems. Lancet.

[3] Ron A.M.D., Sparvoli F., Pueyo J.J., Bazile D. (2017). Editorial: Protein Crops: Food and Feed for the Future. Front. Plant Sci..

[4] Demmig-Adams B., López-Pozo M., Polutchko S.K., Fourounjian P., Stewart J.J., Zenir M.C., Adams W.W. (2022). Growth and Nutritional Quality of Lemnaceae Viewed Comparatively in an Ecological and Evolutionary Context. Plants.

[5] Appenroth K.-J., Sree K.S., Bog M., Ecker J., Seeliger C., Böhm V., Lorkowski S., Sommer K., Vetter W., Tolzin-Banasch K. (2018). Nutritional Value of the Duckweed Species of the Genus *Wolffia* (Lemnaceae) as Human Food. Front. Chem..

[6] Muller T., Cournoyer A., Bazinet L. (2025). Emerging potentials of duckweed (*Lemnaceae*): From composition to protein uses in food and nutraceuticals—A review. Food Res. Int..

[7] Venkatachalam K., Phongthai S., Puttha R., Wongsa J., Charoenphun N. (2025). *Wolffia globosa* as an Emerging Plant-Based Protein Source for Functional and Nutraceuticals. Foods.

[8] Sirison J., Ruangsomboon S., Jongput B., Aue-umneoy D., Tongsri P. (2025). Cultivation of *Wolffia globosa* and its application in functional food development. Sci. Rep..

[9] Yadav N.K., Patel A.B., Debbarma S., Priyadarshini M.B., Priyadarshi H. (2024). Characterization of Bioactive Metabolites and Antioxidant Activities in Solid and Liquid Fractions of Fresh Duckweed (*Wolffia globosa*) Subjected to Different Cell Wall Rupture Methods. ACS Omega.

[10] Seephua N., Boonarsa P., Li H., Thammapat P., Siriamornpun S. (2025). Nutritional Composition and Bioactive Profiles of Farmed and Wild Watermeal (*Wolffia globosa*). Foods.

[11] Yadav N.K., Patel A.B., Debbarma S., Priyadarshini M.B., Kumar G., Baidya S., Upadhyay A.D. (2024). Characterization of phenolic compounds in watermeal (*Wolffia globosa*) through LC-ESI-QTOF-MS/MS: Assessment of bioactive compounds, in vitro antioxidant and anti-diabetic activities following different drying methods. Food Meas..

[12] Monthakantirat O., Chulikhit Y., Maneenet J., Khamphukdee C., Chotritthirong Y., Limsakul S., Punya T., Turapra B., Boonyarat C., Daodee S. (2022). Total Active Compounds and Mineral Contents in Wolffia globosa. J. Chem..

[13] World Health Organization Dementia Fact Sheet. https://www.who.int/news-room/fact-sheets/detail/dementia.

[14] Tiwari S., Yadav S.K., Kumari M., Dhakal T., Puranik N. (2026). Neuropeptides in the Management of Alzheimer’s Disease: From Pathophysiology to Therapeutic Opportunities. Int. J. Mol. Sci..

[15] Prince M., Wimo A., Guerchet M., Ali G.-C., Wu Y.-T., Prina M. (2015). World Alzheimer Report 2015: The Global Impact of Dementia: An Analysis of Prevalence, Incidence, Cost and Trends.

[16] Nichols E., Steinmetz J.D., Vollset S.E., Fukutaki K., Chalek J., Abd-Allah F., Abdoli A., Abualhasan A., Abu-Gharbieh E., Akram T.T. (2022). Estimation of the global prevalence of dementia in 2019 and forecasted prevalence in 2050: An analysis for the Global Burden of Disease Study 2019. Lancet Public Health.

[17] Hardy J., Selkoe D.J. (2002). The amyloid hypothesis of Alzheimer’s disease: Progress and problems on the road to therapeutics. Science.

[18] Tamagno E., Guglielmotto M., Vasciaveo V., Tabaton M. (2021). Oxidative Stress and Beta Amyloid in Alzheimer’s Disease. Which Comes First: The Chicken or the Egg?. Antioxidants.

[19] Pooladgar P., Sakhabakhsh M., Taghva A., Soleiman-Meigooni S. (2022). Donepezil Beyond Alzheimer’s Disease? A Narrative Review of Therapeutic Potentials of Donepezil in Different Diseases. Iran. J. Pharm. Res..

[20] Tan C.-C., Yu J.-T., Wang H.-F., Tan M.-S., Meng X.-F., Wang C., Jiang T., Zhu X.-C., Tan L. (2014). Efficacy and safety of donepezil, galantamine, rivastigmine, and memantine for the treatment of Alzheimer’s disease: A systematic review and meta-analysis. J. Alzheimers Dis..

[21] Williams R.J., Spencer J.P.E. (2012). Flavonoids, cognition, and dementia: Actions, mechanisms, and potential therapeutic utility for Alzheimer disease. Free Radic. Biol. Med..

[22] Sharma A., Rudrawar S., Bharate S.B., Jadhav H.R. (2024). Recent advancements in the therapeutic approaches for Alzheimer’s disease treatment: Current and future perspective. RSC Med. Chem..

[23] Arias-Sánchez R.A., Torner L., Navarro B.F. (2023). Polyphenols and Neurodegenerative Diseases: Potential Effects and Mechanisms of Neuroprotection. Molecules.

[24] Ashwinia P., Subhashb B., Amolb M., Kumarac D., Atmaramd P., Ravindraa K. (2025). Comprehensive investigation of multiple targets in the development of newer drugs for the Alzheimer’s disease. Acta Pharm. Sin. B.

[25] Rebas E., Rzajew J., Radzik T., Zylinska L. (2020). Neuroprotective Polyphenols: A Modulatory Action on Neurotransmitter Pathways. Curr. Neuropharmacol..

[26] Godos J., Carota G., Caruso G., Micek A., Frias-Toral E., Giampieri F., Brito-Ballester J., Rodríguez Velasco C.L., Quiles J.L., Battino M. (2025). Molecular mechanisms underlying the neuroprotective effects of polyphenols: Implications for cognitive function. Excli J..

[27] Das S., Majumder T., Sarkar A., Mukherjee P., Basu S. (2020). Flavonoids as BACE1 inhibitors: QSAR modelling, screening and in vitro evaluation. Int. J. Biol. Macromol..

[28] Rezai-Zadeh K., Ehrhart J., Bai Y., Sanberg P.R., Bickford P., Tan J., Shytle R.D. (2008). Apigenin and luteolin modulate microglial activation via inhibition of STAT1-induced CD40 expression. J. Neuroinflamm..

[29] Kowalczyk A., Tuberoso C.I.G., Jerković I. (2025). Experimental Evidence of Caffeic Acid’s Neuroprotective Activity in Alzheimer’s Disease: In Vitro, In Vivo, and Delivery-Based Insights. Medicina.

[30] Jang S., Johnson R.W. (2011). Can Consuming Flavonoids Restore Old Microglia to their Youthful State?. Nutr. Rev..

[31] Szwajgier D., Borowiec K., Pustelniak K. (2017). The Neuroprotective Effects of Phenolic Acids: Molecular Mechanism of Action. Nutrients.

[32] de la Monte S.M., Wands J.R. (2008). Alzheimer’s Disease is Type 3 Diabetes—Evidence Reviewed. J. Diabetes Sci. Technol..

[33] Wee A.S., Nhu T.D., Khaw K.Y., Tang K.S., Yeong K.Y. (2023). Linking Diabetes to Alzheimer’s Disease: Potential Roles of Glucose Metabolism and Alpha-Glucosidase. Curr. Neuropharmacol..

[34] Nyakayo O., Extension K.P. (2025). The Synergistic Effects of Combination Therapies: Medicinal Plants and Pharmaceuticals. Newport Int. J. Public Health Pharm..

[35] Chaachouay N. (2025). Synergy, Additive Effects, and Antagonism of Drugs with Plant Bioactive Compounds. Drugs Drug Candidates.

[36] Inthachat W., Chantong B., Pitchakarn P., Takoon C., Karinchai J., Suttisansanee U., Temviriyanukul P. (2024). Enhancing Therapeutic Efficacy of Donepezil, an Alzheimer’s Disease Drug, by *Diplazium esculentum* (Retz.) Sw. and Its Phytochemicals. Pharmaceuticals.

[37] Xiao L., Tang J., Tan H., Xie Y., Wang S., Xie L., Wu D. (2024). Efficacy and safety of ginkgo biloba extract combined with donepezil hydrochloride in the treatment of Chinese patients with vascular dementia: A systematic review meta-analysis. Front. Pharmacol..

[38] Montgomery D.C. (2017). Design and Analysis of Experiments.

[39] Bezerra M.A., Santelli R.E., Oliveira E.P., Villar L.S., Escaleira L.A. (2008). Response surface methodology (RSM) as a tool for optimization in analytical chemistry. Talanta.

[40] Kongsung S., Inthachat W., Chantong B., Suttisansanee U., On-Nom N., Chupeerach C., Thangsiri S., Pitchakarn P., Temviriyanukul P. (2024). Box-Behnken Design-Based Optimization of Phytochemical Extraction from *Diplazium esculentum* (Retz.) Sw. Associated with Its Antioxidant and Anti-Alzheimer’s Properties. Molecules.

[41] Calderon O.H., Vega R. (2020). Optimization of the extraction of phenolic compounds and antioxidant activity from the roots of Waltheria ovata using the response surface methodology. Food Res. Int..

[42] Kiani H.S., Ali B., Al-Sadoon M.K., Al-Otaibi H.S., Ali A. (2023). LC-MS/MS and GC-MS Identification of Metabolites from the Selected Herbs and Spices, Their Antioxidant, Anti-Diabetic Potential, and Chemometric Analysis. Processes.

[43] Morris G.M., Huey R., Lindstrom W., Sanner M.F., Belew R.K., Goodsell D.S., Olson A.J. (2009). AutoDock4 and AutoDockTools4: Automated docking with selective receptor flexibility. J. Comput. Chem..

[44] Sarno T.A., Talib L.L., Joaquim H.P., Bram J.M., Gattaz W.F., Forlenza O.V. (2017). Protein Expression of BACE1 is Downregulated by Donepezil in Alzheimer’s Disease Platelets. J. Alzheimers Dis..

[45] Ayaz M., Sadiq A., Junaid M., Ullah F., Ovais M., Ullah I., Ahmed J., Shahid M. (2019). Flavonoids as Prospective Neuroprotectants and Their Therapeutic Propensity in Aging Associated Neurological Disorders. Front. Aging Neurosci..

[46] Hamsalakshmi, Alex A.M., Marappa M.A., Joghee S., Chidambaram S.B. (2022). Therapeutic benefits of flavonoids against neuroinflammation: A systematic review. Inflammopharmacology.

[47] Burton G.W., Traber M.G. (1990). Vitamin E: Antioxidant activity, biokinetics, and bioavailability. Annu. Rev. Nutr..

[48] Ramsay R.R., Tipton K.F. (2017). Assessment of Enzyme Inhibition: A Review with Examples from the Development of Monoamine Oxidase and Cholinesterase Inhibitory Drugs. Molecules.

[49] Hetényi C., van der Spoel D. (2006). Blind docking of drug-sized compounds to proteins with up to a thousand residues. FEBS Lett..

[50] Salmaso V., Moro S. (2018). Bridging Molecular Docking to Molecular Dynamics in Exploring Ligand-Protein Recognition Process: An Overview. Front. Pharmacol..

[51] Hollingsworth S.A., Dror R.O. (2018). Molecular Dynamics Simulation for All. Neuron.

[52] Ye F., Liang Q., Li H., Zhao G. (2015). Solvent effects on phenolic content, composition, and antioxidant activity of extracts from florets of sunflower (*Helianthus annuus* L.). Ind. Crops Prod..

[53] Mokrani A., Madani K. (2016). Effect of solvent, time and temperature on the extraction of phenolic compounds and antioxidant capacity of peach (*Prunus persica* L.) fruit. Sep. Purif. Technol..

[54] Dai J., Mumper R.J. (2010). Plant Phenolics: Extraction, Analysis and Their Antioxidant and Anticancer Properties. Molecules.

[55] Rodrigues L.G., Kemény Z., Berkó S., Kovács A., Vági E.M. (2026). Supercritical carbon dioxide extraction of grape pomace and skin: Enhancing resource efficiency. J. Supercrit. Fluids.

[56] Slimani C., Fadil M., Rais C., Ullah R., Iqbal Z., Santanatoglia A., Caprioli G., Benjelloun M., Lazraq A., Bouyahya A. (2025). Optimization of phenolic compounds extraction and antioxidant activity from Moroccan Crocus sativus L. by-products using predictive modeling and Box-Behnken design. Microchem. J..

[57] Ou B., Hampsch-Woodill M., Prior R.L. (2001). Development and Validation of an Improved Oxygen Radical Absorbance Capacity Assay Using Fluorescein as the Fluorescent Probe. J. Agric. Food Chem..

[58] Prior R.L., Wu X., Schaich K. (2005). Standardized Methods for the Determination of Antioxidant Capacity and Phenolics in Foods and Dietary Supplements. J. Agric. Food Chem..

[59] Shahidi F., Ambigaipalan P. (2015). Phenolics and polyphenolics in foods, beverages and spices: Antioxidant activity and health effects—A review. J. Funct. Foods.

[60] Yan R., Vassar R. (2014). Targeting the β secretase BACE1 for Alzheimer’s disease therapy. Lancet Neurol..

[61] Ghosh A.K., Osswald H.L. (2014). BACE1 (β-secretase) inhibitors for the treatment of Alzheimer’s disease. Chem. Soc. Rev..

[62] Shirkhan F., Mirdamadi S., Akbari-adergani B., Aryaee H. (2026). A review on natural enzyme inhibitors: An effective approach to control postprandial hyperglycemia in type II diabetes. Nat. Prod. Bioprospect..

[63] Birari R.B., Bhutani K.K. (2007). Pancreatic lipase inhibitors from natural sources: Unexplored potential. Drug Discov. Today.

[64] Choi S.-H., Hur J.-M., Yang E.-J., Jun M., Park H.-J., Lee K.-B., Moon E., Song K.-S. (2008). β-secretase (BACE1) inhibitors from *Perilla frutescens* var. *acuta*. Arch. Pharm. Res..

[65] DeRango-Adem E.F., Blay J. (2021). Does Oral Apigenin Have Real Potential for a Therapeutic Effect in the Context of Human Gastrointestinal and Other Cancers?. Front. Pharmacol..

[66] Chou T.-C. (2010). Drug combination studies and their synergy quantification using the Chou–Talalay method. Cancer Res..

[67] Murad H.A.S., Moawadh M.S., Alzahrani A., Alkathiri A.S., Almutairi A., Alhassoun M.I., Alniwaider R.A., Habib A.H., Sain Z.M., Rafeeq M.M. (2024). Computational identification of promising therapeutics via BACE1 Targeting: Implications for Alzheimer’s disease. Cell. Mol. Biol..

[68] Trott O., Olson A.J. (2010). AutoDock Vina: Improving the speed and accuracy of docking with a new scoring function, efficient optimization, and multithreading. J. Comput. Chem..

[69] Sadeghi M., Seyedebrahimi S., Ghanadian M., Miroliaei M. (2024). Identification of cholinesterases inhibitors from flavonoids derivatives for possible treatment of Alzheimer’s disease: In silico and in vitro approaches. Curr. Res. Struct. Biol..

[70] Khan M.T.H., Orhan I., Şenol F.S., Kartal M., Şener B., Dvorská M., Šmejkal K., Šlapetová T. (2009). Cholinesterase inhibitory activities of some flavonoid derivatives and chosen xanthone and their molecular docking studies. Chem. Biol. Interact..

[71] Zhang Q., Yan Y. (2023). The role of natural flavonoids on neuroinflammation as a therapeutic target for Alzheimer’s disease: A narrative review. Neural Regen. Res..

[72] Wasserman A.H., Venkatesan M., Aguirre A. (2020). Bioactive Lipid Signaling in Cardiovascular Disease, Development, and Regeneration. Cells.

[73] Takala R., Ramji D.P., Choy E. (2023). The Beneficial Effects of Pine Nuts and Its Major Fatty Acid, Pinolenic Acid, on Inflammation and Metabolic Perturbations in Inflammatory Disorders. Int. J. Mol. Sci..

[74] Calder P.C. (2009). Polyunsaturated fatty acids and inflammatory processes: New twists in an old tale. Biochimie.

[75] Acosta K., Sree K.S., Okamoto N., Koseki K., Sorrels S., Jahreis G., Watanabe F., Appenroth K.-J., Lam E. (2024). Source of Vitamin B_12_ in plants of the Lemnaceae family and its production by duckweed-associated bacteria. J. Food Compos. Anal..

[76] Xu J., Shen Y., Zheng Y., Smith G., Sun X.S., Wang D., Zhao Y., Zhang W., Li Y. (2021). Duckweed (Lemnaceae) for potentially nutritious human food: A review. Food Rev. Int..

[77] Rosales T.K.O., Silva P.B.V.d., Oliveira P.A., Zanetti M.V., Santos H.A., Fabi J.P. (2025). Nanoencapsulated flavonoids for enhanced modulation of the microbiota-gut-brain axis and neurodegenerative add-on therapy. Trends Food Sci. Technol..

[78] Fang Z., Bhandari B. (2010). Encapsulation of polyphenols—A review. Trends Food Sci. Technol..

[79] Hao M., Tan X., Liu K., Xin N. (2026). Nanoencapsulation of nutraceuticals: Enhancing stability and bioavailability in functional foods. Front. Nutr..

[80] Chan H.Y.E., Bonini N.M. (2000). *Drosophila* models of human neurodegenerative disease. Cell Death Differ..

[81] Pandey U.B., Nichols C.D. (2011). Human disease models in *Drosophila melanogaster* and the role of the fly in therapeutic drug discovery. Pharmacol. Rev..

[82] Maggiore A., Casale A.M., Toscanelli W., Cappucci U., Rotili D., Grieco M., Gagné J.-P., Poirier G.G., d’Erme M., Piacentini L. (2022). Neuroprotective Effects of PARP Inhibitors in *Drosophila* Models of Alzheimer’s Disease. Cells.

[83] Osterwalder T., Yoon K.S., White B.H., Keshishian H. (2001). A conditional tissue-specific transgene expression system using inducible GAL4. Proc. Natl. Acad. Sci. USA.

[84] Nichols C.D., Becnel J., Pandey U.B. (2012). Methods to Assay *Drosophila* Behavior. J. Vis. Exp..

[85] Piper M.D.W., Partridge L. (2018). *Drosophila* as a model for ageing. Biochim. Biophys. Acta Mol. Basis Dis..

[86] Najmi A., Javed S.A., Bratty M.A., Alhazmi H.A. (2022). Modern Approaches in the Discovery and Development of Plant-Based Natural Products and Their Analogues as Potential Therapeutic Agents. Molecules.

[87] Inui T., Wang Y., Pro S.M., Franzblau S.G., Pauli G.F. (2012). Unbiased evaluation of bioactive secondary metabolites in complex matrices. Fitoterapia.

[88] Sirichai P., Kittibunchakul S., Thangsiri S., On-Nom N., Chupeerach C., Temviriyanukul P., Inthachat W., Nuchuchua O., Aursalung A., Sahasakul Y. (2022). Impact of Drying Processes on Phenolics and In Vitro Health-Related Activities of Indigenous Plants in Thailand. Plants.

[89] Chupeerach C., Temviriyanukul P., Thangsiri S., Inthachat W., Sahasakul Y., Aursalung A., Wongchang P., Sangkasa-ad P., Wongpia A., Polpanit A. (2022). Phenolic Profiles and Bioactivities of Ten Original Lineage Beans in Thailand. Foods.

[90] Inthachat W., Promyos N., Suttisansanee U., Chamchan R., Thangsiri S., Chantong B., Pitchakarn P., Temviriyanukul P. (2025). Microwave-assisted optimization extraction for green chili paste and investigation of its alpha-glucosidase inhibitory activity with molecular docking and molecular dynamics simulation for anti-diabetic potential. J. Agric. Food Res..

[91] Kemsawasd V., Thangsiri S., Sahasakul Y., Aursalung A., Inthachat W., Temviriyanukul P., Kittibunchakul S., Suttisansanee U. (2026). Evaluation of the underutilized Malpighia glabra L. fruits as a future functional food: Nutritional composition, phenolic profile, biological activities, and synergistic effects with pharmaceutical drugs. Food Funct..

[92] Wannasaksri W., On-Nom N., Chupeerach C., Temviriyanukul P., Charoenkiatkul S., Suttisansanee U. (2021). In Vitro Phytotherapeutic Properties of Aqueous Extracted Adenia viridiflora Craib. towards Civilization Diseases. Molecules.

[93] Kittibunchakul S., Inthachat W., Sahasakul Y., Boonjoong T., Suttisansanee U., Thangsiri S., Pitchakarn P., Temviriyanukul P., Kemsawasd V. (2026). Nutritional and metabolomic analysis of Selaginella argentea with in vitro and network pharmacology evidence of Alzheimer’s disease-preventive activity. Future Foods.

[94] Temviriyanukul P., Kittibunchakul S., Trisonthi P., Kunkeaw T., Inthachat W., Siriwan D., Suttisansanee U. (2022). Mangifera indica ‘Namdokmai’ Prevents Neuronal Cells from Amyloid Peptide Toxicity and Inhibits BACE-1 Activities in a Drosophila Model of Alzheimer’s Amyloidosis. Pharmaceuticals.

