# Supplementary material for: Optimization and Metabolomic Profiling of Wolffia globosa Extract Reveal Multitarget Neuroprotective Activity Against Alzheimer’s Disease with In Vitro, In Silico, and In Vivo Validation
Source: Int J Mol Sci. 2026 Sep 17;27(18):8266. doi: 10.3390/ijms27188266 (PMC13607432; doi:10.3390/ijms27188266)
Supplement: Supplementary file 1 [file ijms-27-08266-s001.zip › ijms-4501655-supplementary.pdf]

A

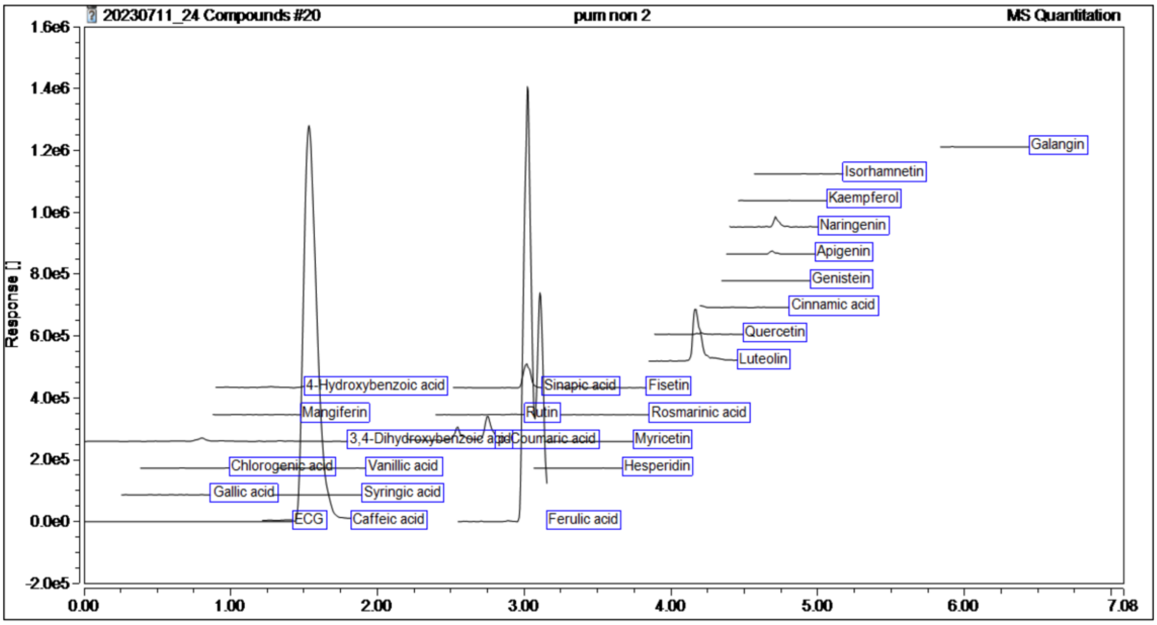

B

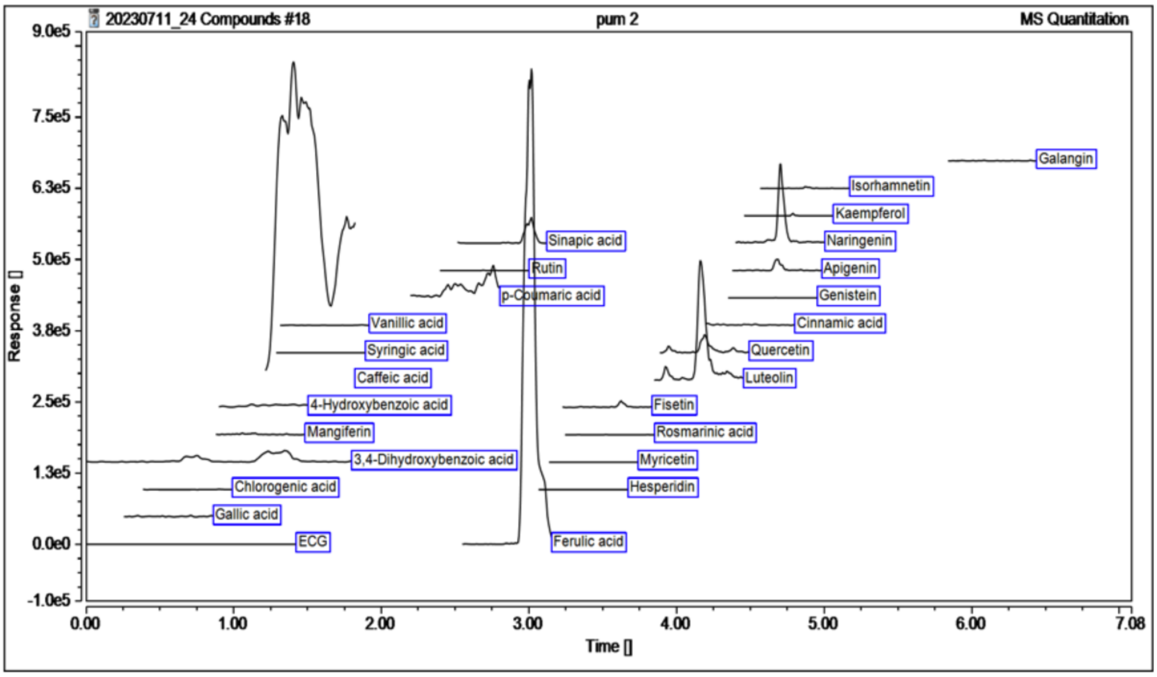

Figure S1. LC–MS/MS chromatographic profile of *W. globosa* extract revealed distinct phenolic profiles under non-acid (**A**) and acid hydrolysis (**B**) conditions.

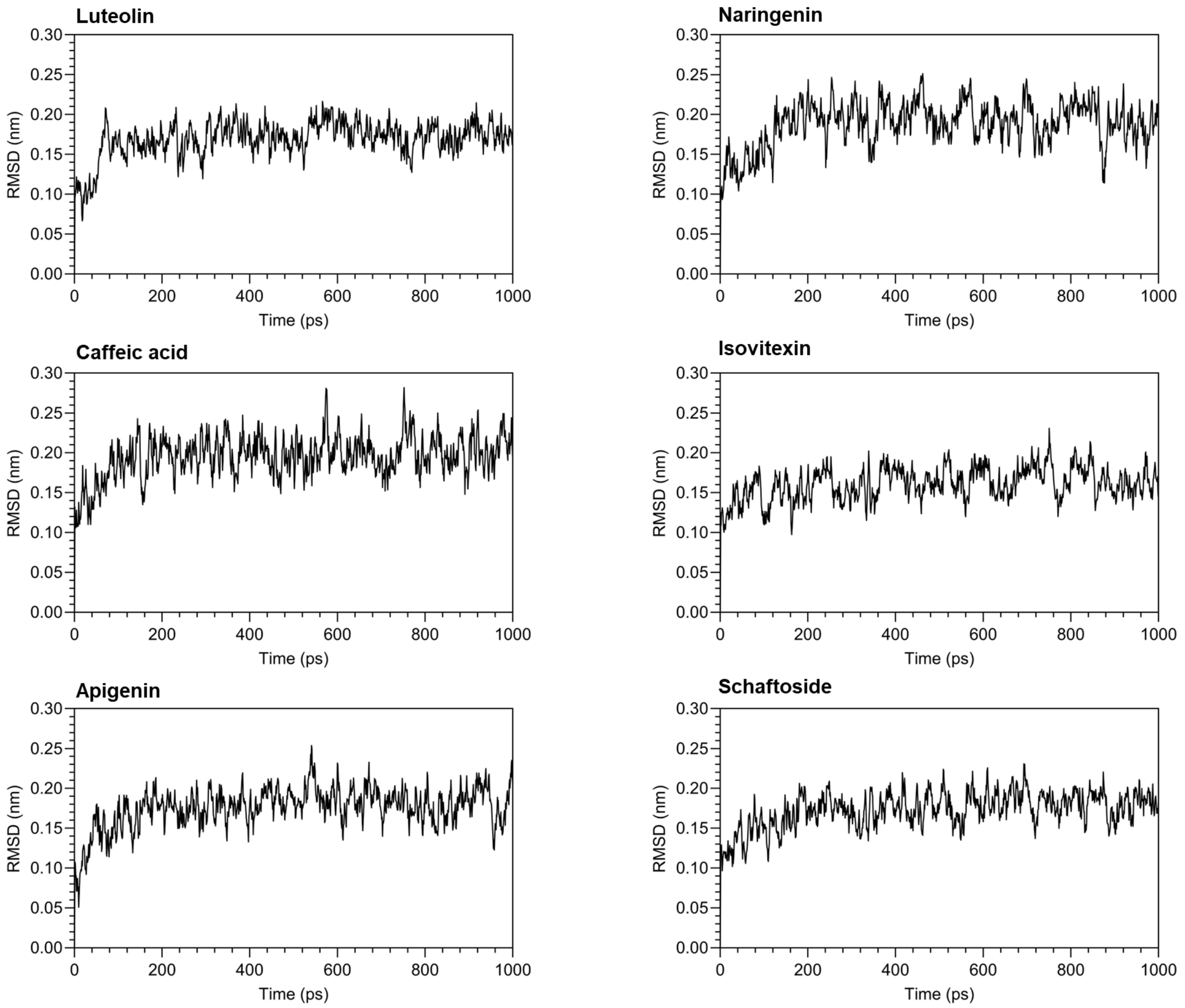

Figure S2. RMSD value from molecular dynamic (MD) simulation of luteolin, caffeic acid, apigenin, naringenin, isovitexin and Schaftoside against AChE.

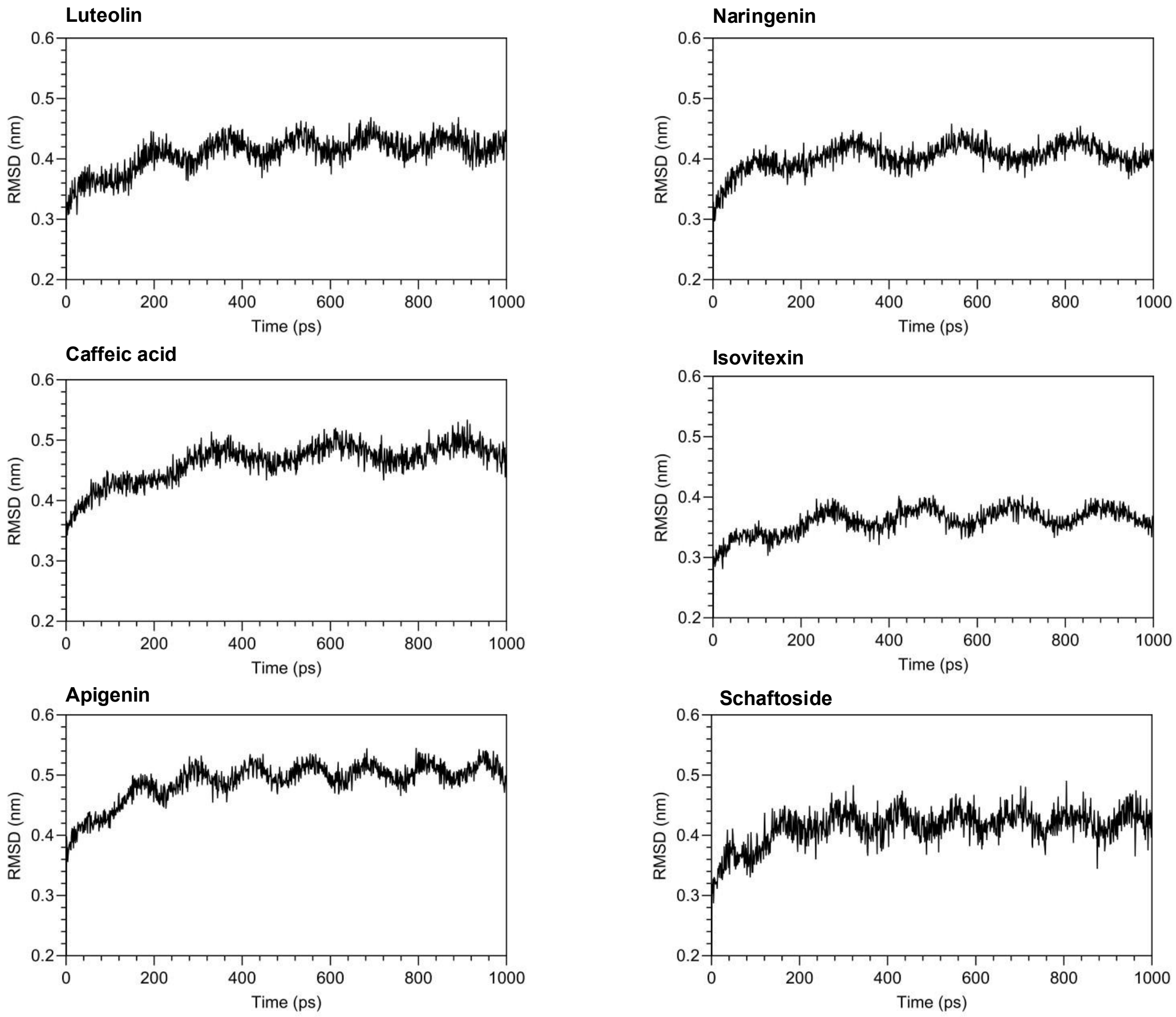

Figure S3. RMSD value from molecular dynamic (MD) simulation of luteolin, caffeic acid, apigenin, naringenin, isovitexin and schaftoside against BChE.

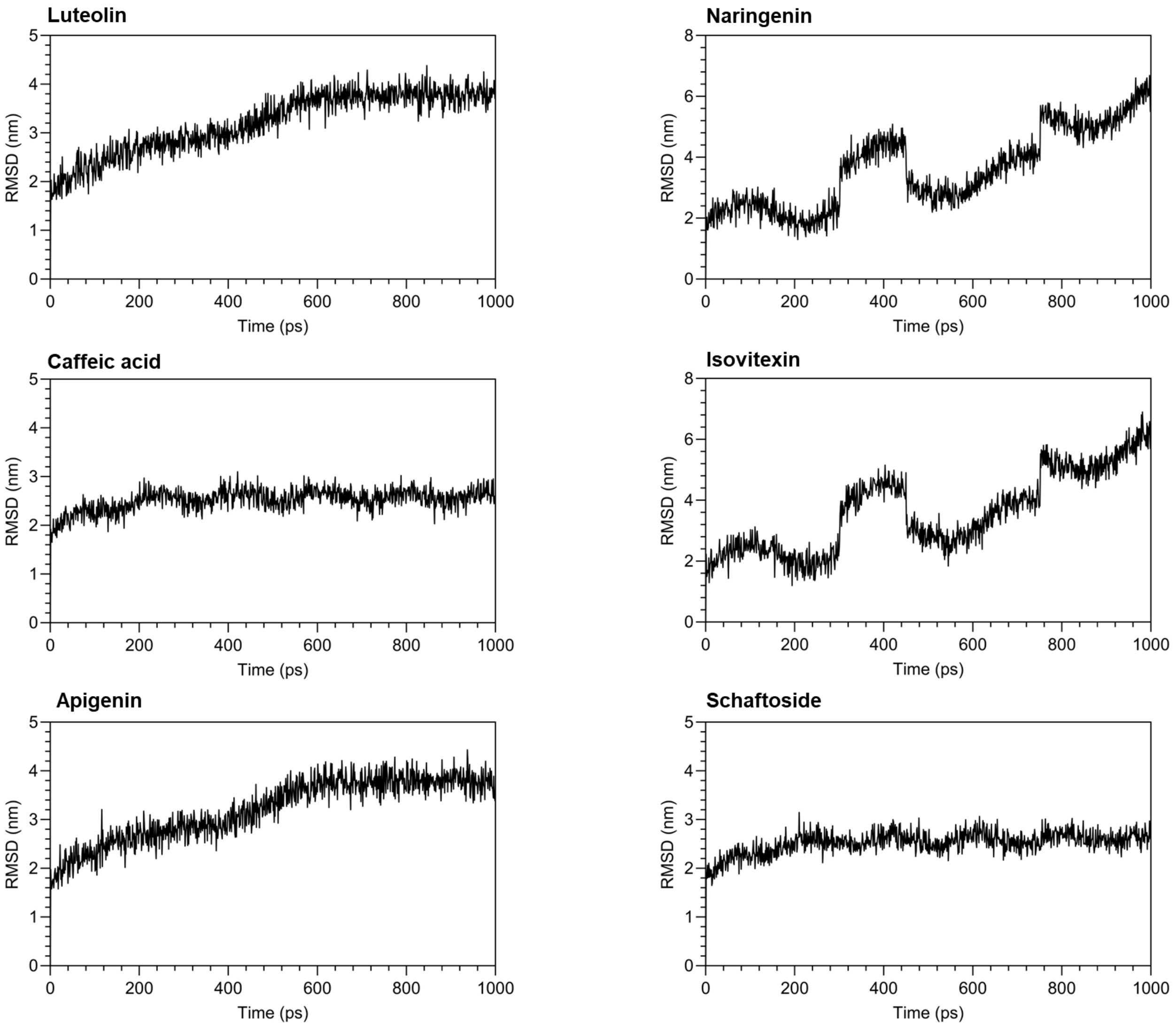

Figure S4. RMSD value from molecular dynamic (MD) simulation of luteolin, caffeic acid, apigenin, naringenin, isovitexin and schaftoside against BACE-1.

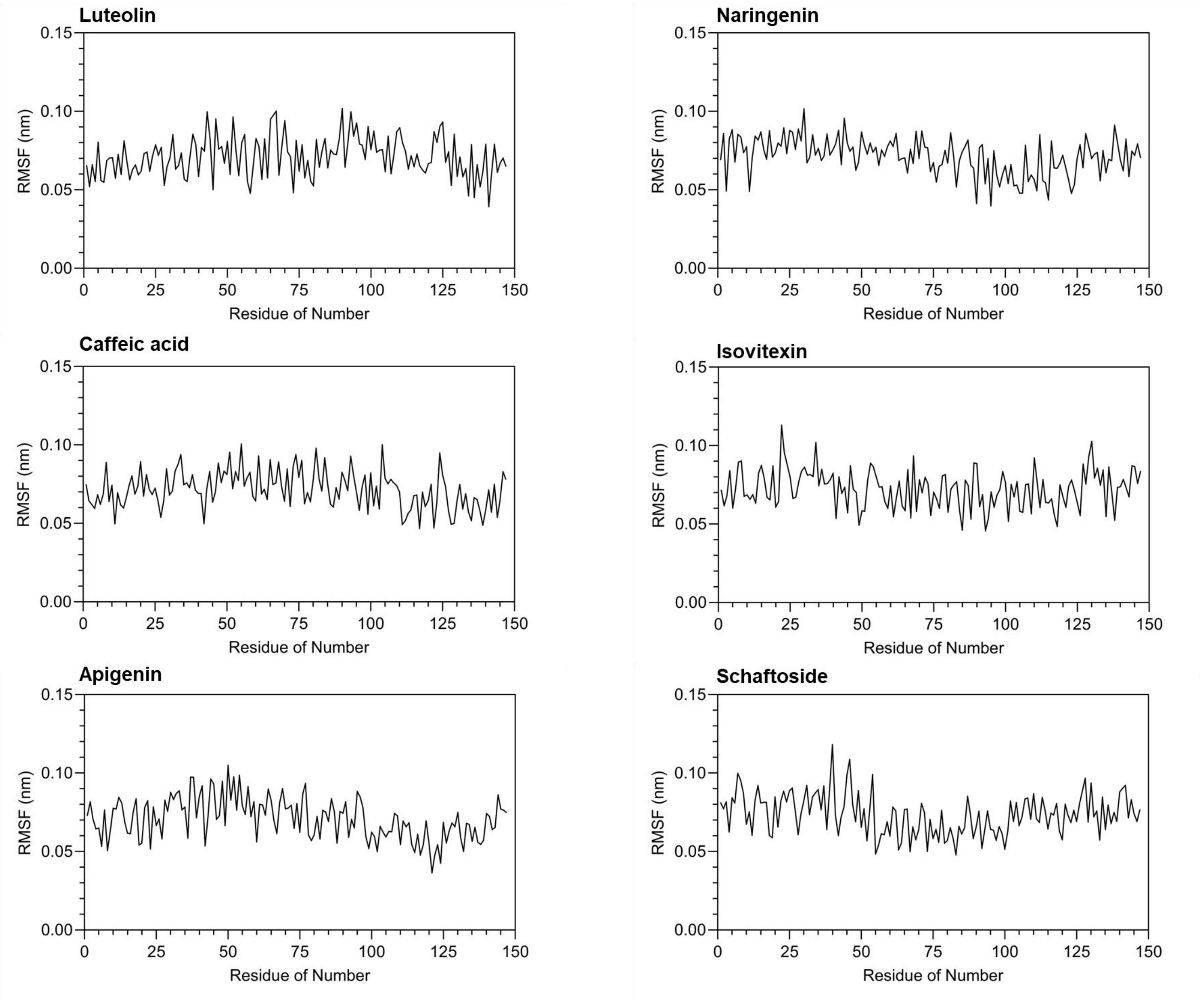

Figure S5. RMSF value from molecular dynamic (MD) simulation of luteolin, caffeic acid, apigenin, naringenin, isovitexin and schaftoside against AChE.

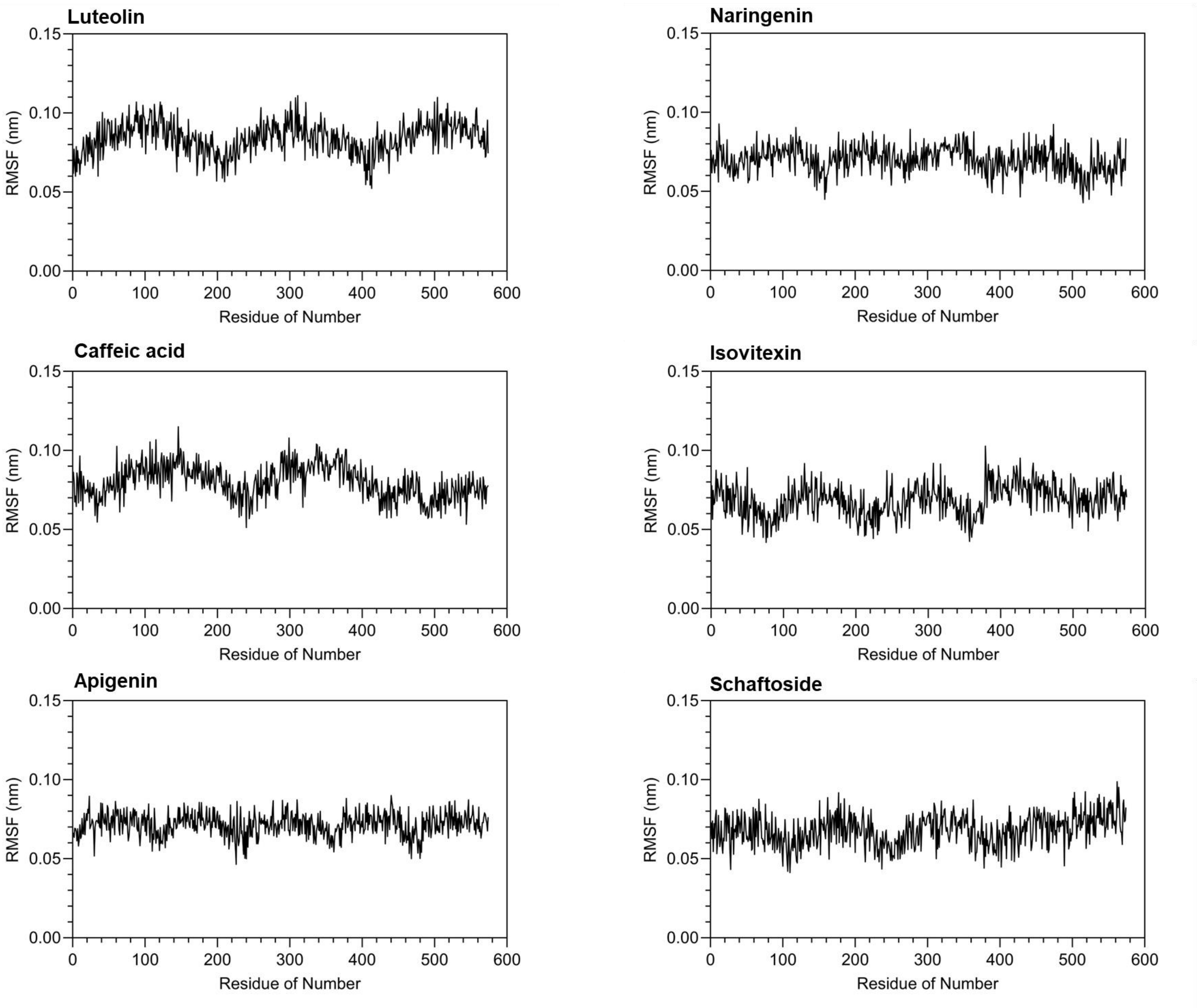

Figure S6. RMSF value from molecular dynamic (MD) simulation of luteolin, caffeic acid, apigenin, naringenin, isovitexin and schaftoside against BChE.

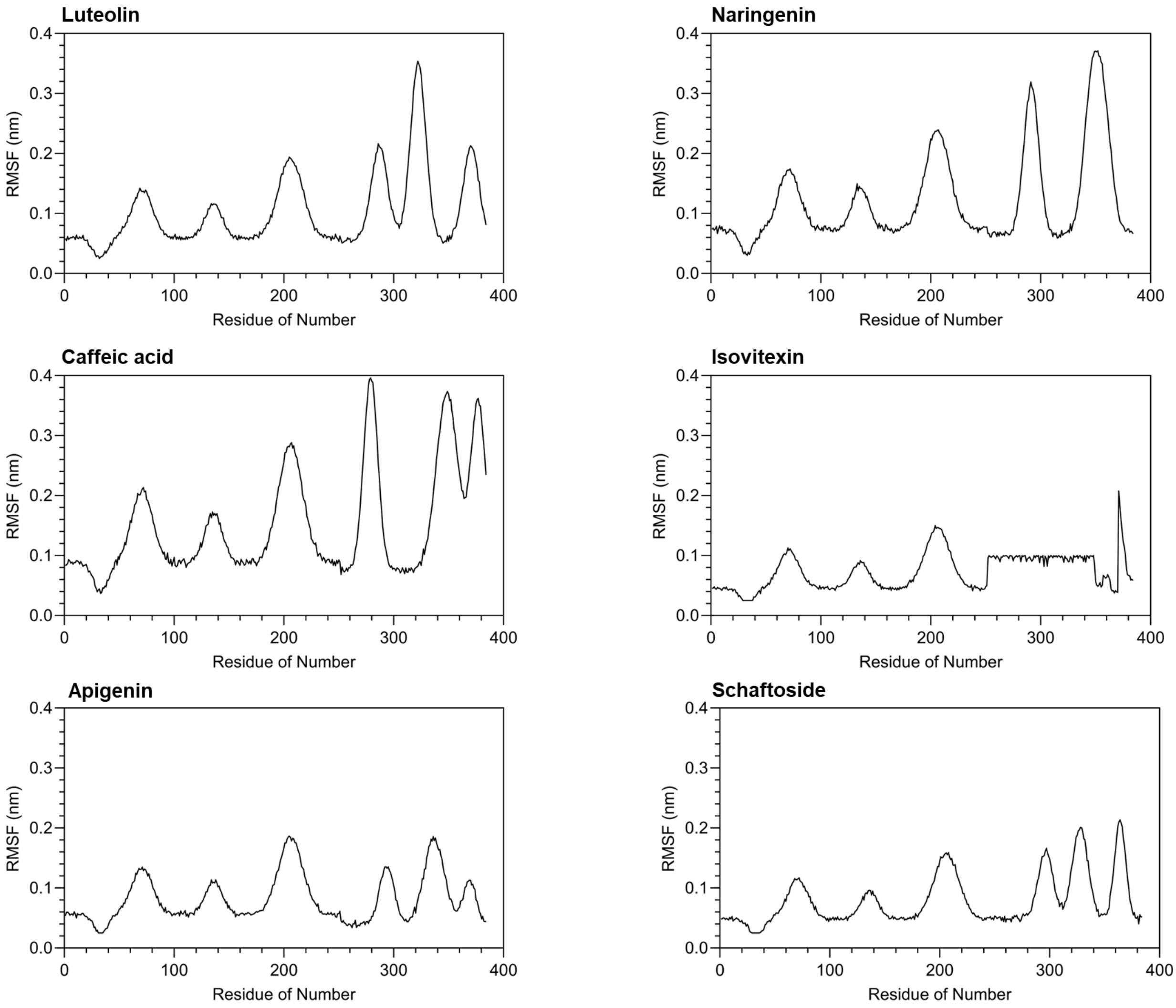

Figure S7. RMSF value from molecular dynamic (MD) simulation of luteolin, caffeic acid, apigenin, naringenin, isovitexin and schaftoside against BACE-1.

A

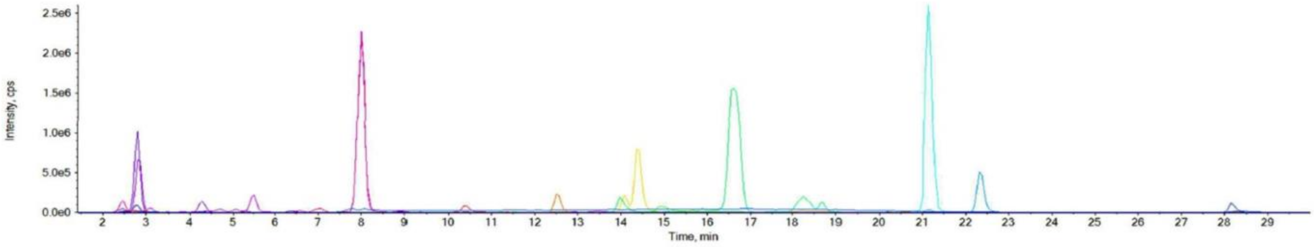

B

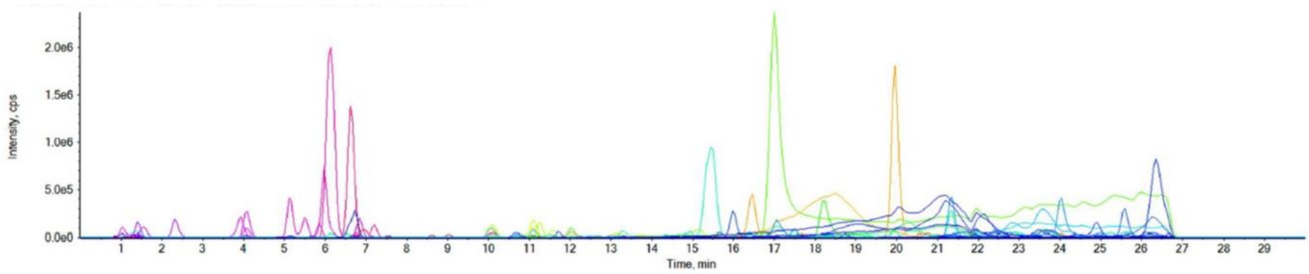

Figure S8. Base peak chromatogram of *W. globosa* extract obtained from HPLC-QTOF-MS/MS. Mass spectrometric data were acquired in both negative (**A**) and positive (**B**) electrospray ionization (ESI) modes over an m/z range of 100–1000.

Table S1. Inhibitory activity of optimized *W. globosa* extract (WGE) against α-Amylase, α-Glucosidase and Lipase.

**Enzymes**	**% Inhibition (2 mg/mL)**	**Half maximal inhibitory concentration IC_{50} (mg/mL)**
α-Amylase	15.46 ± 0.44	ND
α-Glucosidase	ND	Not determined
Lipase	ND	Not determined

ND; Non detected
